# Methods of analysis for survival outcomes with time-updated mediators, with application to longitudinal disease registry data

**DOI:** 10.1177/09622802221107104

**Published:** 2022-06-16

**Authors:** Kamaryn T Tanner, Linda D Sharples, Rhian M Daniel, Ruth H Keogh

**Affiliations:** 1 Department of Medical Statistics, 4906 London School of Hygiene and Tropical Medicine, UK; 2 Division of Population Medicine, 2112 Cardiff University, UK

**Keywords:** Mediation, survival analysis, longitudinal data, simulation study

## Abstract

Mediation analysis is a useful tool to illuminate the mechanisms through which an exposure affects an outcome but statistical challenges exist with time-to-event outcomes and longitudinal observational data. Natural direct and indirect effects cannot be identified when there are exposure-induced confounders of the mediator-outcome relationship. Previous measurements of a repeatedly-measured mediator may themselves confound the relationship between the mediator and the outcome. To overcome these obstacles, two recent methods have been proposed, one based on path-specific effects and one based on an additive hazards model and the concept of exposure splitting. We investigate these techniques, focusing on their application to observational datasets. We apply both methods to an analysis of the UK Cystic Fibrosis Registry dataset to identify how much of the relationship between onset of cystic fibrosis-related diabetes and subsequent survival acts through pulmonary function. Statistical properties of the methods are investigated using simulation. Both methods produce unbiased estimates of indirect and direct effects in scenarios consistent with their stated assumptions but, if the data are measured infrequently, estimates may be biased. Findings are used to highlight considerations in the interpretation of the observational data analysis.

## 1 Introduction

Mediation analysis is used to statistically explore the possible mechanisms through which a treatment or exposure affects an outcome. To achieve this, the analysis attempts to decompose the total effect of the exposure on the outcome into an indirect effect and a direct effect. The indirect effect is the part of the total effect that is realised by the exposure acting on the mediator and that mediator then acting on the outcome. The direct effect captures the portion of the total effect that does not act via the mediator. Through this decomposition we hope to gain insight about the process through which an effect occurs. We refer readers to VanderWeele^1^ for a thorough overview of mediation analysis.

Most methods for mediation analysis have focused on a single mediator and a continuous or binary outcome, though extensions to more complex settings are emerging. In this paper, we are interested in the setting where the outcome is time to an event and the mediator is a time-dependent variable for which repeated measurements are available. The repeatedly-measured nature of the mediator allows us to focus on a process that evolves over time. For example, consider a situation where the exposure is the onset of a condition that causes a progressive elevation in a biomarker and that high levels of this biomarker lead to increased risk of death. One challenge that arises is that the mediator measurement from one time point may confound the association between the mediator measured at a later time point and the outcome. This is an example of exposure-induced confounding of the mediator-outcome association and, for identification, many methods assume this type of confounding does not exist. Time-to-event outcomes pose another difficulty as survival for a given time is a post-exposure confounder of the association between a mediator measured at a later time and subsequent survival (i.e. survival is required to take a mediator measurement) and, therefore, the exposure affects the mediator both directly and indirectly via survival time.

Vansteelandt et al.^2^ recently proposed a method for mediation analysis in this setting by estimating the combination of path-specific effects where the exposure first acts upon the repeatedly-measured mediator. In other words, this method estimates how the effect of the exposure on the outcome is mediated via the entire longitudinal mediator process. As their approach also accommodates time-varying mediator-outcome confounders, it is suitable for situations where multiple mediators may exist. A second mediation method for this setting was proposed by Aalen et al.^3^ and is based on dynamic path analysis using the additive hazards model. As this method requires that control for confounding be made using only covariate measurements taken prior to the exposure, it is restricted to settings without time-varying confounders.

In Buse et al.^4^ the method proposed by Vansteelandt et al.^2^ was applied to data from a randomised controlled trial (the LEADER trial) to identify possible mediators of the effect of treatment on the risk of cardiovascular events. In another application, this method was used to quantify the indirect effect of treatment on the risk of a composite kidney disease outcome via several candidate mediators.^5^ In analyses such as these based on data from randomised controlled trials, control for confounding of the exposure-outcome and exposure-mediator relationships is rendered unnecessary via the randomisation and there is a clearly defined starting time for each individual at the time of randomisation. To the best of our knowledge, to date the methods of Vansteelandt et al.^2^ and Aalen et al.^3^ have only been applied to data from randomised controlled trials. Our focus is on the use of these methods to address mediation questions using observational data. When working with observational data, such as a registry dataset, control for confounding can only be accomplished via adjustment with measured covariates and in cases where the exposure is onset of a condition, there is no natural time zero for comparison of the exposed and unexposed. Further, measurements of time-updated variables are taken on a schedule designed for long-term data collection as opposed to targeting a specific research question. We discuss these issues in our motivating example, which is based on data from the UK Cystic Fibrosis Registry dataset.

Our primary aim is to more thoroughly evaluate two methods available for mediation analysis in the setting of a repeatedly-measured mediator and a time-to-event outcome: the method of Vansteelandt et al.^2^ and the method of Aalen et al.^3^ Although Vansteelandt et al.^2^ provide a basic simulation illustrating their approach for cases with and without direct and indirect effects in an appendix, we are not aware of any extensive simulation study of their method or any simulation study of the method of Aalen. Here, we use simulation to look both at scenarios where we expect good performance as well as at scenarios that may challenge the methods. Further, we apply these methods to analyse mediation in cystic fibrosis-related diabetes. This is the first application of causal mediation methods to the UK Cystic Fibrosis Registry dataset and may motivate further research into mechanisms of disease progression. The paper is organised as follows. Section 2 introduces the motivating application: cystic fibrosis-related diabetes. In Section 3, a brief introduction to causal mediation analysis is provided as well as descriptions of the two mediation analysis methods studied. We present a simulation study assessing the performance of the two methods with a focus on bias in Section 4. Section 5 contains an analysis of the UK Cystic Fibrosis Registry dataset to investigate mediation of the effect of cystic fibrosis-related diabetes on survival. We conclude with a discussion in Section 6.

## 2 Motivating application: Cystic fibrosis-related diabetes

This study is motivated by the setting of cystic fibrosis (CF) and the desire to better understand the mechanisms associated with mortality. CF is a genetic, life-shortening disease that affects more than 10,500 people in the UK^6^ and approximately 100,000 people worldwide.^7^ It is characterised by a progressive loss of lung function and most people with CF die from respiratory failure.^8^ Although there is no cure for CF, improved care and early diagnosis have led to substantial improvements in life expectancy, with a median predicted survival age for babies born today in the UK of 50.6 years.^6^ The increasing lifespan of people with CF is concomitant with an increased risk of co-morbidities, the most common being CF-related diabetes (CFRD). CFRD has been shown to be associated with an increased risk of mortality^9–12^ but the mechanisms for this effect are not well understood. One hypothesis is based on the association of CFRD with worse pulmonary function.^13–15^ Using causal mediation analysis, we aim to quantify how much of the effect of CFRD on survival is mediated through lung function.

We use data from the UK CF Registry, which holds data on more than 12,000 people with CF representing over 99% of the CF population in the UK. This registry dataset contains demographic information, genotype, and time-updated measures of pulmonary function, bacterial infections and other health indicators.^16^ Data are systematically collected at approximately annual routine monitoring visits conducted at specialist CF centres.

## 3 Causal mediation

### 3.1 Background

We begin by outlining some concepts for the setting of a single mediator and a non-time-to-event outcome. Many mediation analyses based on counterfactuals will aim to estimate the natural indirect effect (NIE) and natural direct effect (NDE).^17,18^ In a setting with a binary exposure A , a single mediator M measured once and outcome Y , define $Y_{i}(1)$ and $Y_{i}(0)$ to be the outcome for individual i if the exposure had been set to 1 (exposed) or 0 (unexposed), respectively. We can only observe one of these outcomes for each individual i ; the other is counterfactual. We similarly define $M_{i}(1)$ and $M_{i}(0)$ as the mediator value that would have been seen if i were exposed or unexposed, respectively. Let $Y_{i}(a,M_{i}(a^{*}))$ represent the potential outcome for individual i if their exposure was set to a and the mediator was set to the level it would have taken if the exposure had been $a^{*}$ . Note that a may or may not equal $a^{*}$ . Using this nested counterfactual framework, the NIE and NDE can be defined as:

(1) NIE=E[Y(1,M(1))−Y(1,M(0))]

(2) NDE=E[Y(1,M(0))−Y(0,M(0))]

The NIE captures the change in outcome that would result from fixing the exposure but changing the mediator from the level it would have taken if exposed to the level it would have taken if unexposed. The NDE captures the effect of the exposure on the outcome if the mediator had taken the level it would have taken without exposure. The total causal effect of the exposure on the outcome is the sum of the NIE and NDE. These natural effects can be estimated under the assumption of no unmeasured exposure-outcome, mediator-outcome or exposure-mediator confounding and that there is no exposure-induced mediator-outcome confounding. These are strong assumptions that not even a randomised controlled trial may satisfy. In particular, the assumption of no exposure-induced mediator-outcome confounding is problematic because it requires that no such confounder exists. In other words, being able to measure such a confounder does not allow us to obtain unbiased estimates of the NIE and NDE. Although this assumption will not hold in many settings, it may be reasonable if the mediator is measured a very short time after the exposure.^19^

In the setting studied here involving a repeatedly-measured mediator, survival outcome, and possible time-varying confounding, natural indirect and direct effects cannot be identified. As mentioned in the Introduction, there are problems with exposure-induced mediator-outcome confounding and with survival acting as a post-exposure confounder. In the next sections, we outline the methods of Vansteelandt et al.^2^ and Aalen et al.^3^ which have been developed to overcome these limitations.

### 3.2 Method of Vansteelandt et al. (2019)

Vansteelandt et al.^2^ proposed a mediation analysis method suitable for settings with a time-to-event outcome, time-updated mediator and time-varying confounding of the mediator-outcome association using counterfactual scenarios based on a hypothetical intervention on the mediator. Their proposal infers the effect of the exposure on the outcome through combinations of path-specific effects via the time-updated mediator measurements. A key contribution of this method is that it allows for time-varying mediator-outcome confounders, which could themselves be affected by the exposure.

**Figure 1.**
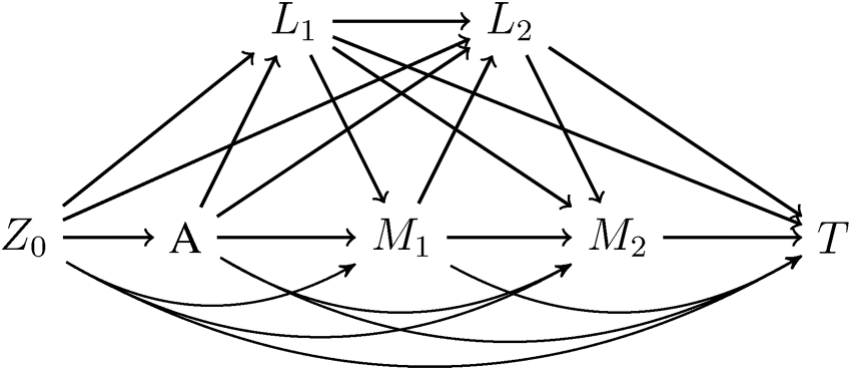
Data-generating mechanism assumed in the method of Vansteelandt for the case of two post-exposure visits. The time-varying confounder $L_{k}$ may influence $M_{k}$ and $M_{k}$ may influence $L_{k+1}$ . Survival to visit k is included in the definition of L . T indicates survival past the second mediator measurement and $Z_{0}$ is the set of baseline covariates, which includes $M_{0}$ and $L_{0}$ .

Consider a situation in which mediators and other time-dependent covariates are observed at regular visit times. The data-generating mechanism and causal ordering for the case of two post-exposure visits is shown in Figure 1. Let A be a binary exposure measured at time t= 0 and k index the visit at which the mediator and other time-updated variables are measured. $M_{k}$ and $L_{k}$ are the values of the mediator and the time-varying confounder(s) at visit k , respectively. $L_{k}$ includes an indicator of whether the person remains at risk at visit k . The set of baseline confounders, which may include $M_{0}$ and $L_{0}$ , are represented by $Z_{0}$ .

**Figure 2.**
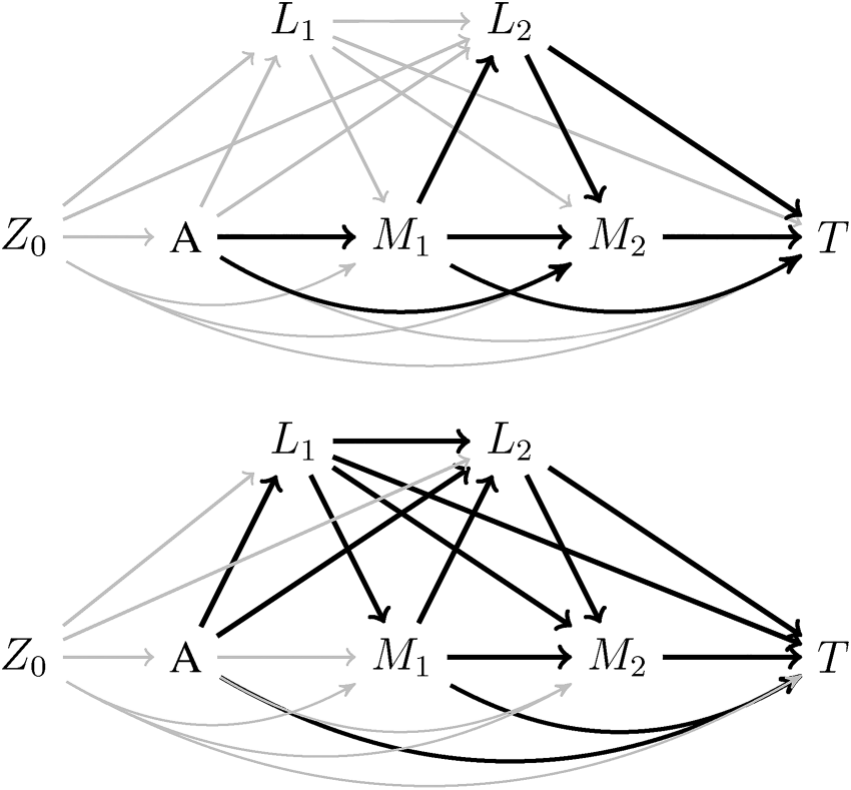
Highlighted path-specific effects. In the top directed acyclic graph (DAG), the path-specific indirect effect of A on T via M is the combination of pathways in black. This is the set of pathways where A first affects M . On the bottom, the combination of pathways in black captures the effect of A on T *not* via M . Note that these pathways may involve M , but only when A affects another variable first. This is referred to as the direct effect.

The indirect effect of the exposure on the outcome via the mediator is taken to be the combination of paths where one or more measurements of the mediator are directly influenced by the exposure and the mediator subsequently affects the outcome. These pathways that make up the indirect effect are shown in black in Figure 2 – upper panel. Conversely, the effect of the exposure on the outcome not via the mediator, referred to as the direct effect, is defined as the combination of paths where the exposure does not directly influence the mediator. These pathways include those in which the exposure first affects L and then L affects M as shown in black in Figure 2 – bottom panel.

Under the method of Vansteelandt, we estimate the survival probabilities $S_{A(a),M(a^{*})}(t)=\mathrm{Pr}\{T(A(a),L(a),M(a^{*}))>t\}$ , where $T(A(a),L(a),M(a^{*}))$ denotes the counterfactual event time had a person’s exposure been set to a and their mediators set to the level they would have taken if the exposure had been $a^{*}$ . For example, for 1<t≤2 this is the probability of survival to time t if all individuals had exposure A=a , time-varying confounders $L_{1}$ remain at the levels they would have taken with exposure A=a and mediator levels $M_{1}$ were set to the levels they would have taken with exposure $A=a^{*}$ if $L_{1}$ had taken the value it would have taken under A=a . Because M and L can affect one another, their counterfactual values rely on all previously set levels. For 2<t≤3 , $L_{2}$ is set to $L_{2}(a,M_{1}(a^{*},L_{1}(a)))$ and $M_{2}$ is set to $M_{2}(a^{*},L_{1}(a),M_{1}(a^{*},L_{1}(a)),L_{2}(a,M_{1}(a^{*},L_{1}(a))))$ . By varying a and $a^{*}$ , three survival functions of interest are estimated: $S_{A(1),M(0)}(t)$ , $S_{A(1),M(1)}(t)$ and $S_{A(0),M(0)}(t)$ and these can be contrasted as differences or ratios to estimate the path-specific effects via M and not via M . We use ratios as this simplifies estimation of the method of Aalen (see next section) and define the IE( t ) and DE( t ) as:

(3) $$IE(t)=S_{A(1),M(1)}(t)/S_{A(1),M(0)}(t)$$

(4) $$DE(t)=S_{A(1),M(0)}(t)/S_{A(0),M(0)}(t)$$

Based on the edge g-formula applied to counterfactuals^20^ and assuming no unmeasured confounding, the causal ordering shown in Figure 1, non-informative censoring and that the causal structure represents a non-parametric structural equation model with independent errors, Vansteelandt et al.^2^ demonstrate that $S_{A(a),M(a^{*})}(t)$ can be identified by:

(5) $$\begin{array}{r} \begin{array}{cc} S_{A(a),M(a^{*})}(t) & =\int f(T>t|T>\lfloor t\rfloor ,{\bar{M}}_{\left\lfloor t\right\rfloor },{\bar{L}}_{\left\lfloor t\right\rfloor },A=a,Z_{0})\\ & \;\times \prod_{s=1}^{\left\lfloor t\right\rfloor }f(M_{s}|T>s,{\bar{L}}_{s},{\bar{M}}_{s-1},A=a^{*},Z_{0})\\ & \;\times \;f(L_{s}|T>s-1,{\bar{L}}_{s-1},{\bar{M}}_{s-1},A=a,Z_{0})\\ & \;\times \;f(Z_{0})dM_{s}dL_{s}dZ_{0} \end{array} \end{array}$$

where ${\bar{M}}_{s}$ ( ${\bar{L}}_{s}$ ) is the history of the mediator (time-varying confounders) up to time s and ⌊t⌋ is the visit time at or before time t . Note that $L_{t}$ contains both the individual’s measured clinical levels and an indicator of whether they remain at risk at time t . Although similar, this is distinct from the mediational g-formula for non-nested counterfactuals.^21^ Repeated regressions are used to estimate $S_{A(a),M(a^{*})}(t)$ . Models are specified for each term in equation (5) and fitted using the observed data. The models are fitted in a sequential way, working backwards from the last visit time to the first. At each step, the outcome of a model is a predicted fitted value between 0 and 1 from a previous model. The final result is an estimated survival probability to time t for each individual, conditional on $Z_{0}$ , and these are averaged to estimate $S_{A(a),M(a^{*})}(t)$ . We refer readers to Vansteelandt et al.^2^ for details of the procedure. Because this method does not assume a particular parametric model, any suitable model may be used in each regression.

### 3.3 Method of Aalen et al. (2020)

Aalen et al.^3^ proposed a mediation analysis method for the special case of a time-to-event outcome and time-updated mediator where control for confounding of the exposure-mediator and exposure-outcome relationships can be achieved using only the set of baseline confounders. When using this method, it is assumed that there are no time-varying confounders of the mediator-outcome association and only a single mediator. A key idea in the method of Aalen is exposure separation.^22,23^ This assumes that the exposure can be separated into two components: one that acts on the mediator process and one that affects survival either directly or through pathways not involving the mediator. Biologically, this means the exposure must be able to be split into separate physiological mechanisms that we could, in theory, manipulate independently of one another.

**Figure 3.**
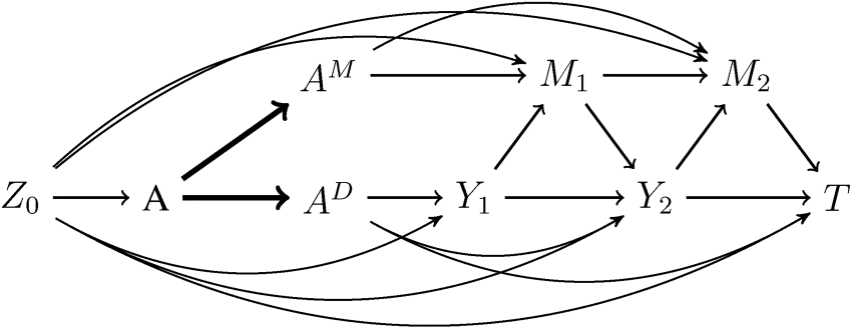
Data-generating mechanism assumed in the method of Aalen for the case of two post-exposure visits. The effect of the exposure A (bold arrows) is split into one component, $A^{M}$ , that affects the mediator and one, $A^{D}$ , that affects survival through other pathways not involving the mediator. $Y_{1},Y_{2}$ indicate survival to visits 1 and 2, respectively and T represents the survival outcome past the second visit. $Z_{0}$ is the set of baseline covariates, which includes $M_{0}$ .

We use the same notation as described in the method of Vansteelandt and introduce $A^{M}$ and $A^{D}$ , which denote the separate components of the exposure A . $A^{M}$ represents the part of the exposure that acts through the mediator process and $A^{D}$ represents the part of the exposure that affects survival via pathways not through the mediator. Define $Y_{t}=I(T>t)$ to be an indicator of survival time T being greater than t . Figure 3 shows the data-generating mechanism for two post-exposure visits. The separation of the exposure into two components is represented by bold arrows. Because individuals will either be exposed or unexposed, we will only observe the scenario where $A=A^{D}=A^{M}$ . However, exposure separation contemplates hypothetical interventions on both $A^{M}$ and $A^{D}$ where $A^{D}$ is not necessarily equal to $A^{M}$ .

The estimand is a survival probability, $\mathrm{Pr}\{T(A^{D}=a,A^{M}=a^{*})>t|Z_{0}\}$ where $T(A^{D}=a,A^{M}=a^{*})$ is the counterfactual survival time given a hypothetical intervention where $A^{D}$ was set to level a and $A^{M}$ was set to level $a^{*}$ . This is estimated using a continuous-time mediational g-formula derived from a linear mediator model and an additive hazards model. Aalen et al.^3^ show how a mediator model that is marginal over the past history of M is specified. The mediator measurement $M_{ij}$ for the $i^{th}$ individual ( i =1, …, n) at the $j^{th}$ event time ( j=1,…,J ) is:

(6) $$M_{ij}=m_{0,j}+\beta_{A,j}A_{i}+\beta_{Z_{0},j}Z_{0,i}+\eta_{ij}$$

where j indexes the unique ordered event times $t_{j}$ , $m_{0,j}$ is the intercept, the error term $\eta_{ij}$ is assumed independent of $A_{i}$ and $Z_{0,i}$ , and $\beta_{A,j}$ and $\beta_{Z_{0},j}$ are coefficients.

An additive hazards model for the hazard of the event at time t conditional on the history of the mediator, the exposure and the baseline covariates is:

(7) $$\lambda_{i}(t|{\bar{M}}_{i,r(t)},A_{i},Z_{0,i})=\alpha_{0,t}+\alpha_{A,t}A_{i}+\alpha_{M,t}M_{i,r(t)}+\alpha_{Z_{0},t}Z_{0,i}$$

where $\alpha_{0,t}$ is the intercept and $\alpha_{A,t}$ , $\alpha_{M,t}$ and $\alpha_{Z_{0},t}$ are coefficients, all of which may be time-dependent. Here, r(t)=k for $t_{k}\leq t<t_{k+1}$ and ${\bar{M}}_{r(t)}$ is the history of the mediator values for times $t\leq t_{r(t)}$ . The model in (7) incorporates an assumption of this method that only the most recent value of the mediator, $M_{r(t)}$ , directly affects the hazard at time t ; the prior mediator history does not impact it. This is shown in Figure 3 by an absence of an arrow from $M_{1}$ to T . Assuming the models in (6) and (7), and using the mediational g-formula, the estimand of interest can be written:

(8) $$\begin{array}{rl} Q(t;A^{D}=a,A^{M}=a^{*},Z_{0}) & :\;=\mathrm{Pr}\{T(A^{D}=a,A^{M}=a^{*})>t|Z_{0}\}\\ & \;\;\;\;\;\;\;\;\;\;\;\;\;\;\;\;\;\;\;=f(t,Z_{0})\times \\ & \exp\;\left\{-a\int_{0}^{t}\alpha_{A,u}\;du-a^{*}\int_{0}^{t}\alpha_{M,u}\beta_{A,r(u)}\;du\right\} \end{array}$$

where $f(t,Z_{0})$ is a function representing the part of the expression that depends only on t and $Z_{0}$ .

The assumptions made in equations (6) and (7) result in the special form of the mediational g-formula in (8). This allows for simple expressions for the IE and DE based on the probabilities $Q(t;A^{D},A^{M},Z_{0})$ :

(9) $$\begin{array}{rl} IE(t) & =Q(t;A^{D}=1,A^{M}=1,Z_{0})/Q(t;A^{D}=1,A^{M}=0,Z_{0})\\ & \;\;\;\;\;\;\;\;\;\;\;\;\;\;\;\;\;\;\;\;\;\;\;\;=\exp\;\left\{-\int_{0}^{t}\alpha_{M,u}\beta_{A,r(u)}\;du\right\} \end{array}$$

(10) $$\begin{array}{rl} DE(t) & =Q(t;A^{D}=1,A^{M}=0,Z_{0})/Q(t;A^{D}=0,A^{M}=0,Z_{0})\\ & \;\;\;\;\;\;\;\;\;\;\;\;\;\;\;\;\;\;\;\;\;\;\;\;=\exp\;\left\{-\int_{0}^{t}\alpha_{A,u}\;du\right\} \end{array}$$

When the direct and indirect effects are defined as ratios, the resulting quantities are independent of $Z_{0}$ .

In the estimation procedure, event times are modelled as a counting process. At each event time, equation (7) is used to regress the change in the counting process onto the mediator and exposure and equation (6) is used to regress the mediator onto the exposure. The integrals in equations (9) and (10) are estimated as cumulative sums of the estimates of the model coefficients, with the integral in equation (10) being the standard cumulative coefficient reported from an additive hazards model. We refer readers to Aalen et al.^3^ and Strohmaier et al.^24^ for a detailed description of their dynamic path analysis approach to estimating the above quantities.

### 3.4 Comparison of the method of Vansteelandt and the method of Aalen

#### 3.4.1 Conceptual considerations

The two approaches outlined above differ fundamentally in their conceptual approach to mediation in a survival context. A key difference is the nature of the counterfactuals. In the method of Vansteelandt, we consider a hypothetical intervention on the mediator to set it to a level that would have been seen if the exposure were different. This could lead to ill-defined effects when the outcome is survival because if the individual would survive longer when exposed ( $A=a^{*}$ ) than unexposed ( A=a ), the level of the mediator when unexposed is undefined after the time of death. Vansteelandt et al.^2^ suggest this can be conceptualised as a hypothetical intervention that sets the mediator to the level the individual would have had if their death had been prevented under the unexposed scenario. As Vansteelandt et al. acknowledge, this may be difficult to envisage. Although we are not required to imagine a mediator value for a person at time t in a scenario in which they would have died before time t , we do need to be able to imagine a mediator value for a person at time t in a hypothetical scenario in which they are alive at time t , but who in reality died before time t . The result is a complication in the interpretation of the mediation results. An alternative interpretation of the Vansteelandt et al. identification result (equation 5) is given by the stochastic randomised intervention approach.^25,26^ Instead of hypothetically intervening on the mediator using a counterfactual scenario had the exposure been a , we can equivalently imagine that the level of the mediator at a given time is set based on a random draw from the distribution of the mediator among surviving individuals with exposure a , conditional on the history of covariates. This assumes that individuals surviving to time t in the exposed and unexposed scenarios are similar given covariate histories. Vansteelandt et al.^2^ point out that although generally survival bias could lead to exposed individuals being different from unexposed individuals, the assumption of no unmeasured common causes of the mediators and time-varying confounders in their framework escapes this issue. The conceptual difficulty of different survival times under exposed/unexposed counterfactual scenarios is avoided entirely in the method of Aalen. In this method, there is no problem because the hypothetical intervention is on the separated exposure variable, not on the mediator. Therefore, we do not need to imagine a counterfactual outcome under exposure a and mediator $M(a^{*})$ where $a\neq a^{*}$ .

The practical value of methods based on nested counterfactuals, which the method of Vansteelandt relies on, has also been discussed more generally.^27,23,28,29^ It involves considering an individual had their exposure been set to one level, but had their mediator been set to the value that would have been seen under a different exposure level. This is not a situation that could ever be observed in practice, which has raised conceptual concerns about the interpretation of the resulting estimands.

Although the exposure separation approach used in the method of Aalen avoids the use of nested counterfactuals, there are also conceptual hurdles involved in exposure separation. Here, physiologically, we must be able to decompose the exposure status into one component that affects survival but not the mediator and one component that affects the mediator but not survival. The independence of these two components is essential; if, for example, the proposed component affecting the mediator also affects survival, the assumptions of the analysis will not be valid. While an imagined exposure separation could correspond to a testable intervention, in practice, it may be difficult or even clinically impossible to individually manipulate the two components separately.

#### 3.4.2 Statistical considerations

Both approaches require that there is no unmeasured confounding of the exposure-outcome, mediator-outcome and exposure-mediator relationships in order to obtain unbiased estimates of the estimands that they target. The method of Aalen requires that this control for confounding be via confounders measured at baseline and expressly forbids the existence of an exposure-induced mediator-outcome confounder. In contrast, the method of Vansteelandt was designed for settings with time-varying mediator-outcome confounders, including those affected by the exposure, and, as long as they can be measured, identification is possible. Another difference is that the method of Vansteelandt does not rely on parametric models in equation (5) for identification. In theory, arbitrary models may be selected for estimation. In the method of Aalen, however, the simplicity of the form of the IE and DE estimands is due to assumptions that the mediator follows a linear model, that the hazard of an event follows an additive hazards model and that only the most recent value of the mediator is necessary to model the hazard.

Despite these fundamental differences between the two approaches, Vansteelandt et al.^2^ describe their approach as ‘a generalisation of dynamic path analysis’. Further, they showed the equivalence of the two approaches when there are no time-varying confounders, the mediator and hazard for the event follow additive models as in equations (6) and (7) and all individuals survive to the first mediator measurement. Under those conditions, using method of Vansteelandt equations (5) to calculate survival probabilities and (3) and (4) to calculate the IE(t) and DE(t), the resulting expressions are equivalent to those obtained from the method of Aalen for IE(t) and DE(t) in equations (9) and (10). This shows a connection between the nested counterfactual approach and the exposure splitting approach under certain conditions.

## 4 Simulation Study

### 4.1 Design

#### 4.1.1 Overview and aims

To expand our understanding of the performance of these two mediation methods in more complex scenarios with a time-to-event outcome and repeatedly measured mediator, we conducted a simulation study. Both methods were evaluated using scenarios where we expected good performance as well as scenarios with data issues commonly found in observational datasets such as time-varying confounding and infrequent measurements of longitudinal variables. To assist other researchers interested in these methods, R code for generating truth data and simulated data as described in this manuscript is available from https://github.com/KamTan/MediationSimulation. Additionally, we use the results of this simulation study to aid in the interpretation of our analysis of the UK CF Registry dataset (see Section 5).

#### 4.1.2 Data-generating mechanisms

**Figure 4.**
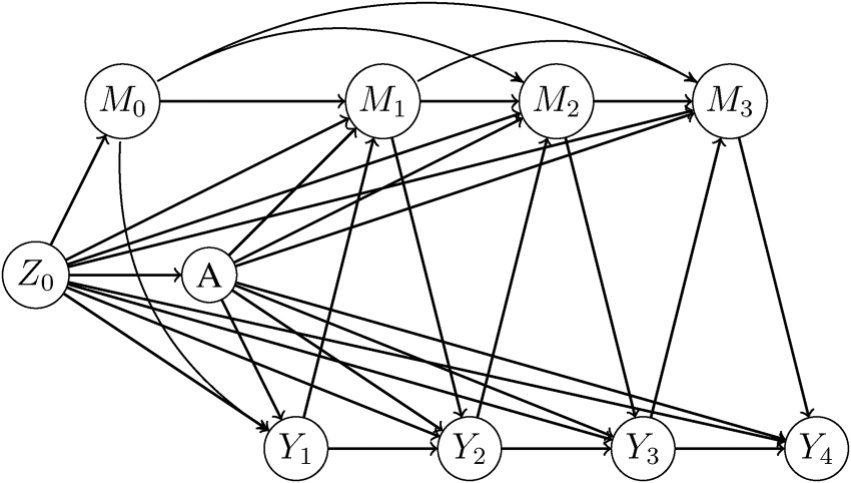
Illustration of the data-generating mechanism. The exposure A occurs at time t=0 and there are baseline measurements of the confounder $Z_{0}$ and mediator $M_{0}$ . $Y_{k}$ is an indicator of survival defined as $Y_{k}=I(T>k)$ . Event times T are generated in waves. The mediator is measured at visit time k for all individuals with T>k .

Several different scenarios were studied and we begin by describing a reference scenario which is consistent with the assumptions of both methods. We consider a setting where there is a binary exposure A at time t= 0 and a binary time-fixed confounder, $Z_{0}$ . The study period is 4 years during which event times T are observed. Individuals who do not have the event are administratively censored at time t= 4. A repeatedly-measured continuous mediator is measured at baseline ( $M_{0}$ ) and at post-exposure visits k= 1,2,3. Note that $M_{0}$ may be affected by $Z_{0}$ . Figure 4 illustrates this reference scenario.

For each scenario considered, three sub-scenarios are studied: (1) both a direct and an indirect effect of the exposure on the outcome are present (“DE+IE”); (2) only an indirect effect is present, meaning there is no path from A to $Y_{t}$ that does not first go through $M_{t}$ (“NoDE”); and (3) only a direct effect is present because the exposure does not affect the mediator (“NoIE”). In Figure 4, removal of all lines from A to $Y_{t}$ corresponds to the NoDE sub-scenario and removal of all lines from A to $M_{t}$ corresponds to the NoIE sub-scenario.

The following steps were used to generate data according to the data generating mechanisms illustrated in Figure 4 for individuals i=1,…,n :

1. Generate $A_{i}$ from a Bernoulli distribution with probability $p_{A}$ =0.5.
2. Generate $Z_{0,i}$ from a Bernoulli distribution with probability $p_{Z_{0}|A}$ , where $p_{Z_{0}|A=1}=$ 0.6 and $p_{Z_{0}|A=0}=$ 0.4. This results in $p_{Z_{0}}=$ 0.5 and is equivalent to simulating the setting where the baseline covariate affects the exposure.
3. Generate values for the longitudinal mediator measurement as random draws from:
(11) $$M_{0,i}∼N\left(\mu =\mu_{m0,i}+\beta_{Z_{0}}Z_{0,i},\;\sigma^{2}=1\right)$$
  and, for t= 1,2,3:
(12) $$M_{t,i}∼N\left(\mu =\mu_{m0,i}+\mu_{m1,i}t+\beta_{Z_{0}}Z_{0,i}+\beta_{A_{t}}A_{i},\;\sigma^{2}=1\right)$$
$\mu_{m0,i}$ , the random intercept and $\mu_{m1,i}$ , the random time slope are generated from a bivariate normal distribution with $\mu_{M}=\left[\begin{array}{c} 2.9\\ 0.0 \end{array}\right]$ and $\Sigma_{M}=\left[\begin{array}{cc} 0.250 & -0.015\\ -0.015 & 0.010 \end{array}\right]$ . The effect of the exposure on the mediator, $\beta_{A_{t}}$ may be time-varying but is constant in the reference scenario.
4. Generate the conditional hazard from:
(13) $$\begin{array}{c} \lambda_{i}(t|A_{i},Z_{0,i},M_{0,i},M_{\lfloor t\rfloor ,i})=\alpha_{0}+\alpha_{A}A_{i}+\alpha_{Z_{0}}Z_{0,i}+\alpha_{M_{\left\lfloor t\right\rfloor }}M_{\lfloor t\rfloor ,i} \end{array}$$
  Here, ⌊t⌋ represents the visit time at or prior to t or 0 for t=0 , $\alpha_{0}$ is the constant baseline hazard, $\alpha_{M_{\left\lfloor t\right\rfloor }}$ is the time-varying effect of the mediator on the hazard, and $\alpha_{A}$ and $\alpha_{Z_{0}}$ are the time-fixed effects of the exposure and baseline confounder on the hazard, respectively. In the reference scenario, $\alpha_{M_{\left\lfloor t\right\rfloor }}$ was constant but it was allowed to have an increasing, step change and decreasing effect in scenarios R1-R3, respectively. An additive hazards model was chosen to generate survival times because the method of Aalen assumes additive hazards.
5. Generate event times T in waves w= 0,1,2,3 for the intervals between visit times [0,1), [1,2), [2,3), [3,4). For individuals still at risk in wave w :
  - Generate $u_{i}∼U(0,1)$
  - Calculate $T_{i}^{\prime }=-\log\;(u_{i})/\lambda_{i}(t|A_{i},Z_{0,i},M_{w,i})$
  - If $T_{i}^{\prime }+w<(w+1)$ , then $T_{i}=T_{i}^{\prime }+w$ . Otherwise, generate a new event time in the next wave.
  - At t= 4, administratively censor all individuals still at risk.
6. Generate an event indicator E equal to 1 if T< 4 and 0 otherwise.

The result is a dataset with values of A , $Z_{0}$ , $M_{0}$ , T , E and $M_{t}$ ( t= 1,2,3) for all simulated individuals. Table 1 in the Supplemental Information provides the parameter values used in data generation. Values of parameters were chosen to yield a positive hazard at all times.

To further probe each method, we considered two additional scenario types: one with infrequent mediator measurements and one with time-varying confounding present. Table 1 provides a summary of the simulation scenarios investigated. To create the additional scenarios, some modification of the data generating procedure was needed as outlined below.

**Table 1.** Listing of all simulation scenarios, the abbreviated name used in the Results section, the percent of simulated individuals experiencing an event prior to time t =4, and the table number where full results are provided in the Supplemental Information for that scenario.

Scenario Type	Description	Scenario name	Events %	Results table
Reference				
	Constant effect of M on hazard	Baseline	87–89	3
	Increasing effect of M on hazard	R1	87–89	–
	Immediate effect of M on hazard	R2	90–92	–
	Delayed effect of M on hazard	R3	84–87	–
Infrequent mediator measurements				
	$\beta_{A_{t}}>0$	F1	87–89	5
	$\beta_{A_{t}}<0$	F2	78–87	6
Time-varying confounders				
	L with $E[\mu_{l1}]=0.5$	L1	84–86	7
	L with $E[\mu_{l1}]=5.0$	L2	87–88	8
	L with $E[\mu_{l1}]=15.0$	L3	91–92	9
	L with $\sigma_{\mu_{l1}}=5.0$	L4	85–87	10
	L with $\sigma_{\mu_{l1}}=10.0$	L5	85–86	11
	L where A affects L	L6	78–82	12

To create scenarios for investigating the impact of an infrequently measured mediator, the above described procedure was adapted to generate mediator values at time intervals of 0.25 (i.e. at times t= 0, 0.25, 0.5, … ,3.75). Event times were generated in waves at the same frequency as the mediator measurements, however, effect estimation was done using only the four mediator measurements at t= 0, 1, 2, 3. The two scenarios generated in this manner differed only in the direction of the effect of A on $M_{1},M_{2},M_{3}$ . The exposure positively affected the mediator in scenario F1 while it negatively affected the mediator in scenario F2.

Finally, to create scenarios with a time-varying confounder, $L_{t}$ , we additionally generate a random intercept $\mu_{l0,i}$ and random time slope $\mu_{l1,i}$ where $\left(\mu_{l0,i},\mu_{l1,i})∼MVN(\mu_{L},\Sigma_{L}\right)$ and i indexes individuals. We assume a causal ordering where $L_{t}$ , measured at the same time as $M_{t}$ , may influence $M_{t}$ and $M_{t}$ may influence $L_{t+1}$ . First, $L_{0}$ and $M_{0}$ are generated as:

(14) $$L_{0,i}∼N\left(\mu =\mu_{l0,i}+\psi_{Z_{0}}Z_{0,i},\;\sigma^{2}=1\right)$$

(15) $$M_{0,i}∼N\left(\mu =\mu_{m0,i}+\beta_{Z_{0}}Z_{0,i}+\beta_{L_{0}}L_{0,i},\;\sigma^{2}=1\right)$$

where $\psi_{Z_{0}}$ is the effect of $Z_{0}$ on $L_{0}$ and $\beta_{L_{0}}$ is the effect of $L_{0}$ on $M_{0}$ . Then, $L_{t}$ and $M_{t}$ are successively generated for t= 1, 2, 3 using:

(16) $$L_{t,i}∼N\left(\mu =\mu_{l0,i}+\mu_{l1,i}t+\psi_{Z0}Z_{0,i}+\psi_{A_{t}}A_{i}+\psi_{M_{t-1}}M_{t-1,i},\;\sigma =1\right)$$

(17) $$M_{t,i}∼N\left(\mu =\mu_{m0,i}+\mu_{m1,i}t+\beta_{Z0}Z_{0,i}+\beta_{A_{t}}A_{i}+\beta_{L_{t}}L_{t,i},\;\sigma =1\right)$$

Event times are generated using the same procedure as described above but the conditional hazard also depends on L as shown below:

(18) $$\lambda_{i}(t|A_{i},Z_{0,i},M_{\lfloor t\rfloor ,i},L_{\lfloor t\rfloor ,i})=\alpha_{0}+\alpha_{A}A_{i}+\alpha_{Z_{0}}Z_{0,i}+\alpha_{M_{\left\lfloor t\right\rfloor }}M_{\lfloor t\rfloor ,i}+\alpha_{L_{\left\lfloor t\right\rfloor }}L_{\lfloor t\rfloor ,i}$$

We generated six scenarios with a time-varying confounder (Table 1). Scenarios L1–L3 are used to study a time-varying confounder generated from different distributions. The impact of greater variability in the random slope is studied with scenarios L4 and L5 and scenario L6 considers the case where A affects the values of L .

#### 4.1.2 Estimands

We focus on two estimands: the indirect effect of the exposure on the outcome via the mediator and the direct effect of the exposure on the outcome not via the mediator (see equations (3) and (4) for the method of Vansteelandt and equations (9) and (10) for the method of Aalen). Total effect (TE) estimates are also reported for completeness. We do not consider proportion mediated as an estimand. Although it is intuitively appealing for quantifying mediation, it tends to have wide confidence intervals^1^ and, when the total effect estimate is small, it becomes unstable as the denominator approaches zero.

#### 4.1.3 Methods

Both the method of Vansteelandt and the method of Aalen were applied to each simulation scenario for effect estimation. We implemented the method of Vansteelandt with an additive hazards model as the simulated event times were generated under this assumption. We use the notation Vansteelandt ${}^{add}$ to remind readers of this choice. A quasi-binomial regression with a logit link was used to model the predictions resulting from each step in the repeated regression estimation procedure. The method of Aalen was implemented with an additive hazards model and linear mediator model. All models for both methods included main effects only. As the implementation provided in Aalen et al.^3^ requires survival to the first visit time, we define our population for both methods to be only those individuals with survival time T≥1 . Note that this does not affect the estimate of indirect effect, which cannot be estimated prior to the first mediator measurement. See the Supplemental Information for details. We included both $Z_{0}$ and $M_{0}$ in all analyses. We refer readers to Landau et al.^30^ in which they concluded that mediation analysis of trial data should include baseline measurements of key variables not only to improve precision but also to avoid potential confounding bias.

#### 4.1.4 Performance measures

The primary performance measure assessed is bias in the estimates of DE( t ), IE( t ), and TE( t ), and we report this at three time points: the times corresponding to the 20th, 50th and 80th percentile of event occurrence (which differ by scenario). Let θ be the true value of the estimand, $\hat{\theta }$ the estimated value of the estimand, $\bar{\theta }$ the mean of ${\hat{\theta }}_{i}$ and $n_{sim}$ the number of simulated datasets. Following Morris et al.^31^ we define the bias and Monte Carlo standard error (MCSE) of the bias as:

$\text{Bias}=\frac{1}{n_{sim}}\sum_{i=1}^{n_{sim}}({\hat{\theta }}_{i}-\theta )$

$\text{MCSE}=\sqrt{\frac{\frac{1}{n_{sim}-1}\sum_{i=1}^{n_{sim}}({\hat{\theta }}_{i}-\theta {)}^{2}}{n_{sim}}}$

Based on the results of several simulation runs, we expect the Var( $\hat{\theta }$ ) to be less than 0.015. Because we require the MCSE to be below 0.005, the minimum number of simulated datasets needed will be 600 given that $n_{sim}=\frac{\text{Var}(\hat{\theta })}{{\text{MCSE}}_{\text{bias}}^{2}}$ . To accommodate cases with slightly greater variance, we use $n_{sim}$ =1000 with $n_{obs}$ =2000 simulated individuals per dataset.

#### 4.1.5 Generation of the true values of the estimands

The true values of the estimands were estimated using a large ( n =3,500,000) simulated dataset that gives stable values following the approach used, for example, by Keogh et al.^32^ For each simulated individual, data were generated for four cases:

-
A=1 with M=M(1)
-
A=0 with M=M(0)
-
A=1 with M=M(0)
-
A=0 with M=M(1)

The same equations used to generate the simulated datasets were used except that the exposure was not affected by $Z_{0}$ or $M_{0}$ , thereby ensuring that there was no exposure-outcome confounding. All simulated individuals with event times prior to the first mediator measurement time were removed. The probability of survival at time t in each of the four cases equals the proportion still at risk at time t as the only censoring in the dataset is administrative censoring at time t= 4. Given these survival probabilities, $S_{A(a),M(a^{*})}(t)$ , the TE, DE and IE at time t are given by:

(19) $$\theta_{TE}(t)=S_{A(1),M(1)}(t)/S_{A(0),M(0)}(t)$$

(20) $$\theta_{DE}(t)=S_{A(1),M(0)}(t)/S_{A(0),M(0)}(t)$$

(21) $$\theta_{IE}(t)=S_{A(1),M(1)}(t)/S_{A(1),M(0)}(t)$$

Note that in the NoDE sub-scenario, $S_{A(1),M(0)}(t)=S_{A(0),M(0)}(t)$ and the total effect equals the indirect effect. Similarly, in the NoIE sub-scenario, the total effect equals the direct effect because $S_{A(1),M(1)}(t)=S_{A(1),M(0)}(t)$ . This is depicted graphically in the Supplemental Information, Figure 1.

#### 4.1.6 Software

R v4.0.2^33^ was used for all analyses and generation of simulated data. We used the timereg^34^ R package for the additive hazards model. For the method of Aalen, we used R code provided in the Supplemental Materials by the authors.^3^

### 4.2 Results

#### 4.2.1 Reference scenario

Using the reference scenario, the estimated TE, DE and IE were approximately unbiased for both methods for all three sub-scenarios (DE+IE, NoDE, NoIE). Full results are shown in Supplemental Information Table 3. The MCSE of all bias estimates was <0.005. The empirical standard error for the method of Aalen was lower in both the DE+IE and NoDE sub-scenarios leading to a relative efficiency greater than one (1.55–2.94) over the method of Vansteelandt (Supplemental Information Table 4).

The reference scenario was extended to study three settings with a time-varying effect of the mediator on the hazard: (R1) an effect that increases over time, (R2) an immediate effect only, and (R3) a delayed effect. Again, both methods produced approximately unbiased effect estimates (percent bias <2% and Monte Carlo standard error <0.005) at all time points for all sub-scenarios (results not shown).

#### 4.2.2 Infrequent mediator measurements

**Figure 5.**
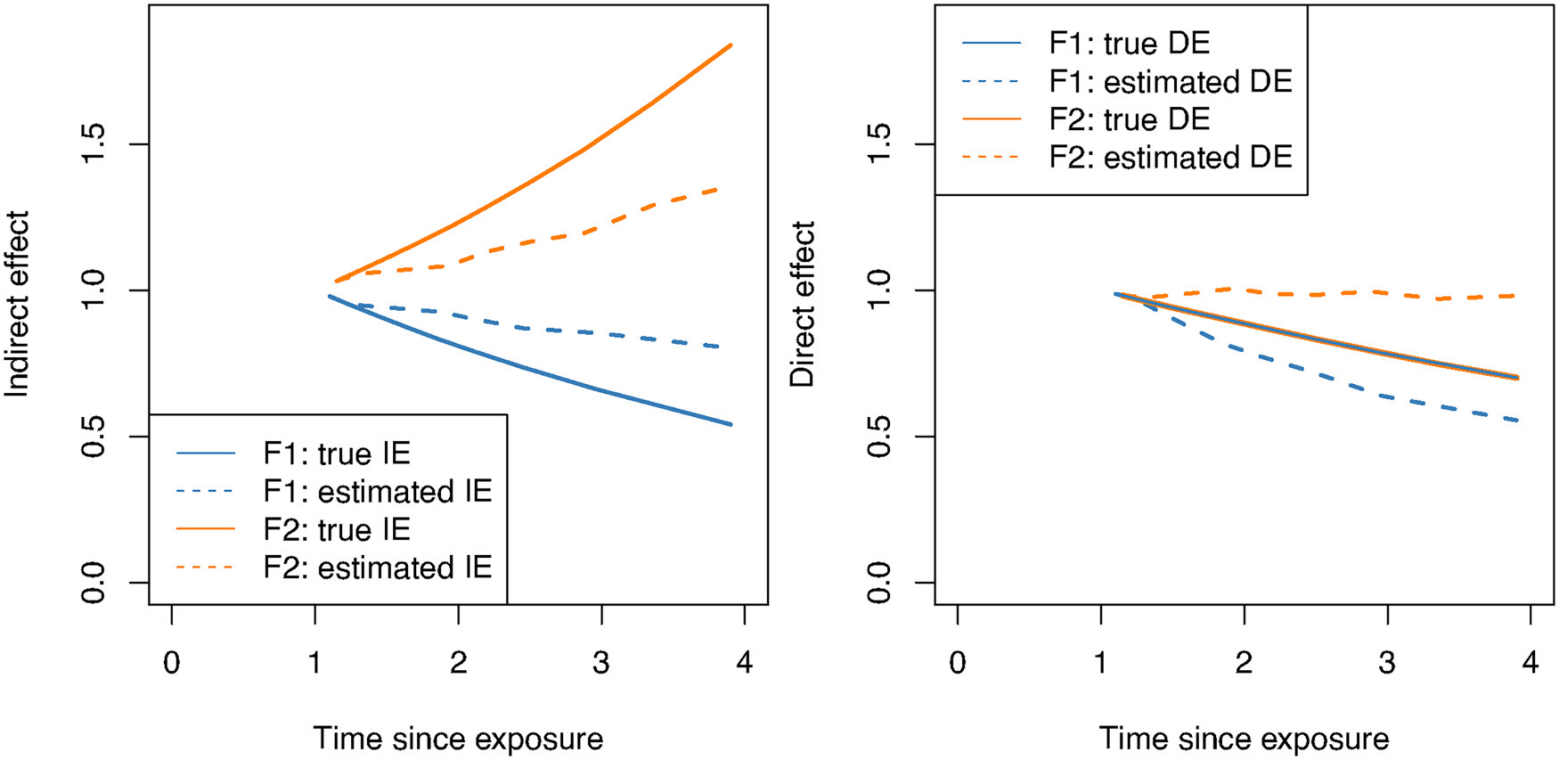
Effect estimates using the method of Vansteelandt when the mediator is infrequently measured. On the left, the true IE (solid line) and the estimated IE (dashed line) are plotted over time. A similar plot for the true DE (solid line) and estimated DE (dashed line) is shown on the right. Estimates and true values for scenario F1 ( $\beta_{A_{t}}=2.0$ ) are in blue and for F2 ( $\beta_{A_{t}}=-2.0$ ) in orange. Exposure occurs at time 0.

For both methods and both scenarios (F1, F2) investigating the impact of infrequent mediator measurements, the estimated indirect effect was closer to 1.0 (no effect) than the true indirect effect in the DE+IE and NoDE scenarios. Figure 5 shows the estimated and true IE (left) and estimated and true DE (right) for scenarios F1–DE+IE and F2–DE+IE, using the method of Vansteelandt. Method of Aalen results were similar. In the F1–DE+IE scenario, both methods over-estimated the IE by 7% when 50% of individuals had experienced an event (time t= 1.66); this increased to 18% by the time 80% of individuals had an event (time t= 2.45). For scenario F2, at the time at which 50% of individuals had an event, the absolute bias in the estimate of IE was − 0.13, equivalent to a percent bias of − 11% in the DE+IE sub-scenario, and was − 0.15 ( − 12%) in the NoDE sub-scenario for both methods.

**Figure 6.**
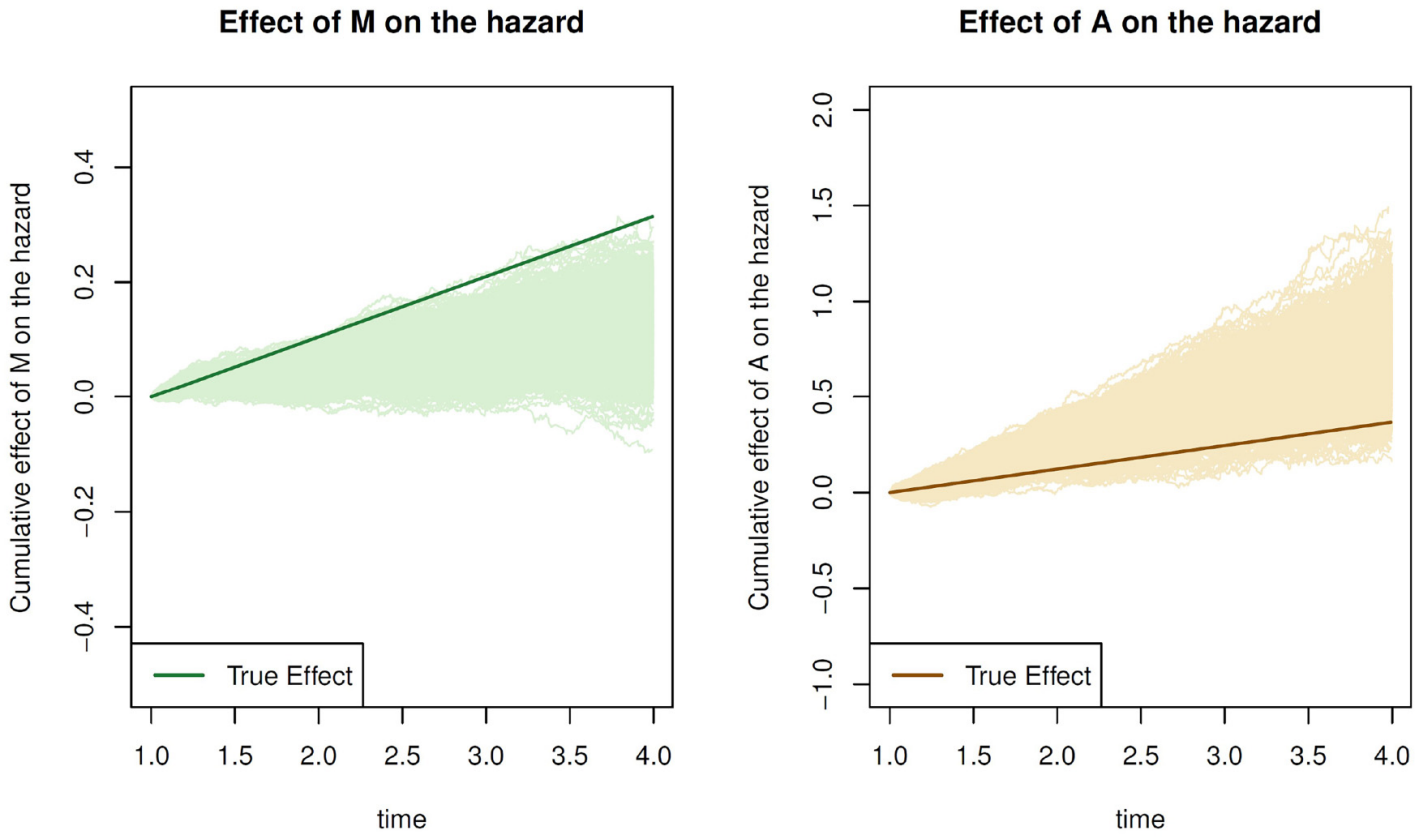
Results from 1000 simulation datasets of scenario F1–DE+IE using the method of Aalen to estimate the parameters from equation 7. On the left, the true value of $\alpha_{M_{t}}^{\prime }$ is shown as a dark green line and estimated values of this parameter in lighter green. On the right, the true value of $\alpha_{A_{t}}^{\prime }$ is shown as a brown line with estimated values in beige.

To better understand the source of this bias, we looked at estimates of the effect of the mediator on the hazard ( $\alpha_{M_{t}}^{\prime }$ in equation 7) and the exposure on the hazard ( $\alpha_{A_{t}}^{\prime }$ in equation 7) using the method of Aalen, which estimates these parameters directly. Figure 6 displays the results for scenario F1–DE+IE. In nearly all of the 1,000 simulation runs, the estimated effect of the mediator on the hazard was lower than the true value but the estimated effect of the exposure on the hazard was greater than the true value. Because $\beta_{A_{t}}^{\prime }$ is positive in scenario F1 and the estimate of $\alpha_{M_{t}}^{\prime }$ is less than the true value, the estimated indirect effect is greater than the true value (see equation 9). In scenario F2, the estimate of $\alpha_{M_{t}}^{\prime }$ was again less than the true value but because $\beta_{A_{t}}^{\prime }$ is negative, the indirect effect estimate was biased downwards.

When the IE was overestimated (underestimated), the corresponding DE was underestimated (overestimated) resulting in an approximately unbiased estimated total effect. Supplemental Information Tables 5 and 6 provide complete results.

#### 4.2.3 Time-varying confounders present

Scenarios L1, L2 and L3 include a time-varying confounder L with mean random slope of 0.5, 5.0 and 15.0, respectively. Both the method of Vansteelandt and the method of Aalen produced approximately unbiased effect estimates for these scenarios. Detailed results are available in the Supplemental Information, Tables 7 to 9. Scenarios L4 and L5 simulated a time-varying confounder with moderate and large variance of the random slope, respectively. The method of Vansteelandt produced unbiased results in these scenarios (Tables 10 and 11 in the Supplemental Information). Some bias was seen in the estimates of DE and IE using the method of Aalen. For scenario L5, the estimate of IE using the method of Aalen when 50% of events had occurred and both a direct and indirect effect were present was underestimated by 2%; IE was underestimated by 8% when 80% of events had occurred. In the final time-varying confounding scenario (L6), the exposure affected the values of $L_{1}$ , $L_{2}$ and $L_{3}$ in the simulated data. In both the DE+IE and NoIE sub-scenarios, bias was seen in the estimates of DE and IE using the method of Aalen (Table 2). By the time 80% of events had occurred, this bias exceeded 10%. Effect estimates from the method of Vansteelandt were approximately unbiased as were effect estimates using the method of Aalen in the NoDE sub-scenario. Full results for scenario L6 are available in Supplemental Information Table 12.

**Table 2.** Bias of effect estimates for scenario L6 with a time-varying covariate that is affected by the exposure. Percent bias is shown beneath the absolute bias in parentheses. Results are shown at times corresponding to the 20th, 50th (median) and 80th percentile of event occurrence for the DE+IE scenario. The Monte Carlo Standard Error was <0.005 for all estimates of absolute bias.

					Absolute bias (percent bias)					
		Truth			Aalen			Vansteelandt ${}^{add}$		
Events	Time	TE	DE	IE	TE	DE	IE	TE	DE	IE
*Both Direct and Indirect Effects*										
20%	1.27	0.94	0.98	0.96	0.00	−0.02	0.02	0.00	0.00	0.00
					(0%)	( − 2%)	(2%)	(0%)	(0%)	(0%)
50%	1.81	0.82	0.93	0.88	0.00	−0.05	0.05	0.00	0.00	0.00
					(0%)	( − 5%)	(6%)	(0%)	(0%)	(0%)
80%	2.71	0.66	0.86	0.76	0.00	− 0.09	0.09	0.00	0.01	0.01
					(0%)	( − 11%)	(12%)	(0%)	(1%)	(1%)

## 5 Application to CF-related diabetes

### 5.1 Methods

Data were obtained from annual review records from the UK CF Registry between 2008 and 2017, inclusive. The study population consisted of all individuals in the UK CF Registry aged 18–60, with known genotype and at least two measurements of forced expiratory volume in 1  second (FEV1%), a key predictor of survival. From this group of 6374 individuals, we further excluded people who were pancreatic insufficient (to ensure positivity) and people who had been diagnosed with CFRD prior to the beginning of follow-up. We excluded these prevalent cases to avoid bias due to unknown duration of disease and focus only on incident cases of CFRD.^35,36^ The resulting cohort consisted of 3708 individuals with 18,693 annual review records.

The exposure was diagnosis of CFRD (Y/N) and the outcome was the composite of death from any cause or lung transplantation. The mediator studied was lung function, measured by FEV1%, a continuous variable. Five baseline confounders were adjusted for in all analyses: gender (M/F), genotype (F508del homozygous Y/N), calendar year, baseline FEV1% and baseline body mass index (BMI). To ensure proper temporal ordering of the data, baseline measurements were taken from the annual review prior to the one where the exposure was assessed and the first mediator measurement was taken from the annual review after exposure assessment. We also adjusted for time-updated measures of BMI (continuous) and respiratory infections (proxied by the number of days in hospital receiving IV antibiotics) when using the method of Vansteelandt. Hospital IV days was categorised into six categories as: 0, 1–7, 8–14, 15–21, 21–28 and >28 days as IV antibiotics are frequently given in week-long courses.

To create the analysis dataset, we assumed that measurements of the exposure, mediator and time-varying covariates were taken at integer-valued ages. For each age, 18–50 years, an age-specific dataset was created comprising all individuals at risk at that age who were either not diagnosed with CFRD or diagnosed with CFRD within the past year. In each age-specific dataset, time was reset to zero when CFRD was or was not diagnosed and age was included as a covariate. In this structure, each individual contributed data as an unexposed person at multiple ages (each age that they were at risk but not diagnosed) but only contributed data as an exposed person at the one age they were first diagnosed, if ever diagnosed. This allows us to make the best use of the longitudinal data in our situation where there is no natural time zero for an unexposed person. More details on the construction of the analysis dataset are available in the Supplemental Information.

Estimates are presented for IE using the same relative survival scale described in the simulation study. Estimates were computed every 0.1 years starting at time t= 0.05 in the method of Vansteelandt analysis. We used a Cox proportional hazards survival model with a linear predictor containing main effects only. Quasi-binomial models with a logit link containing main effects only were used to model the other terms in equation (5). Method of Aalen regressions (also main effects only) were performed at each event time in the study population. Non-parametric bootstrap was used to compute 95% confidence intervals using the percentile method with 500 bootstrap samples and resampling done at the individual level.

### 5.2 Results

**Figure 7.**
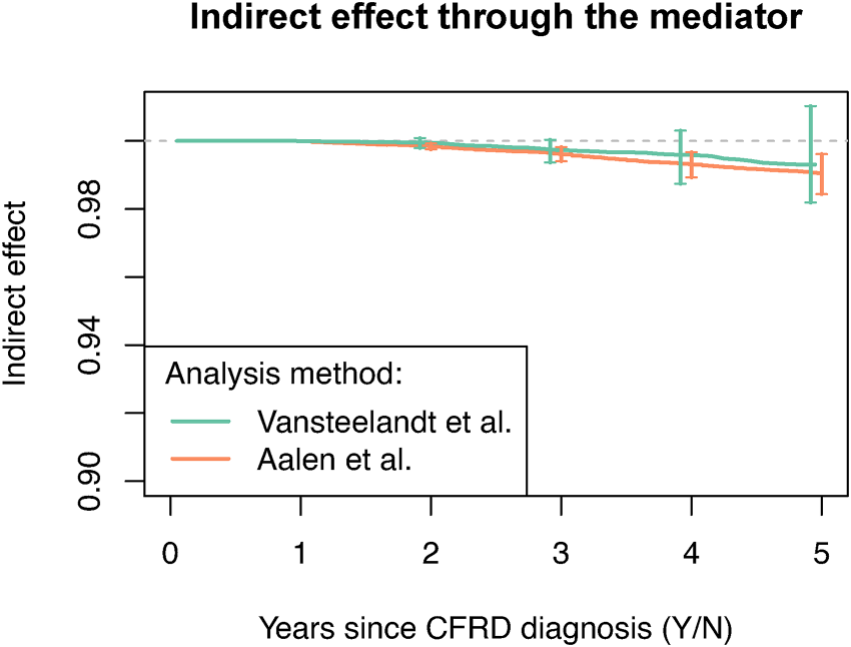
Results from the method of Vansteelandt and the method of Aalen. The indirect effect of CFRD on time to death or lung transplant via FEV1%, the mediator, is shown. Vertical bars at visit times illustrate 95% bootstrap confidence intervals.

Both mediation analysis methods estimated the indirect effect of CFRD on survival via lung function to be modest in size. Figure 7 shows the estimated IE for each mediation method, as a function of time. Using the method of Vansteelandt, the estimated indirect effect increases in magnitude over time, reaching 0.996 at time t= 4 years post-evaluation of CFRD. The interpretation is as follows. Suppose that a random set of individuals in the population had been assigned to have CFRD. The estimand compares (using a ratio) what their survival probability at year 4 would have been had FEV1%, BMI and IV days been set to the levels they would be if they had CFRD versus what it would have been had BMI and IV days been set to the levels they would be if they had CFRD but FEV1% had been set to the level it would have taken had they not had CFRD. Bootstrap confidence intervals for the estimated IE contain 1.0 at all visit times suggesting that there may be no mediation via lung function. The estimated proportion mediated at time t= 4 is 4.5% [95% CI: − 4.3%, 12.8%] with a total effect estimate of 0.918 [95% CI: 0.889, 0.953]. The method of Aalen analysis estimated a slightly greater indirect effect of 0.993 at 4 years post-evaluation of CFRD. Bootstrap confidence intervals of the estimates of IE do not contain 1.0 at times greater than 2 years post-evaluation. This corresponds to an estimated proportion mediated of 7.9% [95% CI: 3.6%, 14.0%] at t= 4 with a total effect estimate of 0.919 [95% CI: 0.888, 0.945].

## 6 Discussion

In this study, we explored two recently proposed methods for mediation analysis in a setting with a time-to-event outcome and a time-updated mediator using a simulation study and in our motivating example of CFRD. Both methods produced approximately unbiased estimates of TE, DE and IE in simulated scenarios consistent with their stated assumptions. When the mediator was measured infrequently or confounding was not controlled for, however, bias was seen in the effect estimates for both methods.

The presence of time-varying confounding of the mediator and outcome is likely in many settings where the mediator is repeatedly-measured over time. An important feature of the method of Vansteelandt et al.^2^ is the ability to identify indirect and direct effects even when time-varying confounding exists. In six simulation scenarios with a time-varying covariate that influenced the mediator process, the method of Vansteelandt returned approximately unbiased effect estimates. Using the method of Aalen, which explicitly assumes that control for confounding can be accomplished with baseline covariates, there is no mechanism to adjust for values of L post-exposure. When the baseline value $L_{0}$ was a good representation of post-exposure values $L_{1}$ , $L_{2}$ and $L_{3}$ , the bias seen was minimal. Greater bias was seen when the post-exposure values of L were affected by the exposure or when the variance of the random slope used to generate values of L was larger. This highlights the importance of understanding whether time-varying confounding exists in a particular setting and, if so, implementing a method that explicitly accounts for it.

Substantial bias was found in the estimates of DE and IE for scenarios with infrequent mediator measurement. For example, this could occur in observational datasets where the mediator is a continuous biomarker measured periodically at infrequent visits. In this case, the analysis incorporates snapshots of the trajectory of the continuous biomarker but survival is affected in continuous time. This is a type of measurement error and it resulted in an attenuation of the effect estimates in our simulation study. Further, the bias accumulated over time. Strohmaier et al.^24^ reported similar results but found that increasing the frequency of the mediator measurements did not necessarily improve estimation of the IE. Rather, less bias was seen when mediator measurements better represented the underlying biological process. Infrequently measured confounders may also contribute to the bias and this bias could be in either direction. Interesting avenues for future research would be to explore methods for mitigating the impacts of measurement error in mediators and infrequent measurements. Correcting for measurement error would require external information about the error, and Aalen et al.^3^ have suggested a calibration approach based on work in VanderWeele^1^ that may be useful when such information is available. A possible solution to the second issue could involve using mixed models or joint models to impute unmeasured longitudinal values of the mediators and covariates.

In the analysis of the UK CF Registry dataset, both methods produced similar effect estimates and found only a small indirect effect of CFRD on survival via lung function. The conclusion is that the majority of the total effect acts through pathways where the exposure does not first affect the mediator process. Because the primary cause of death in CF is respiratory failure and previous studies have shown that CFRD is associated with both increased mortality and decreased lung function, we hypothesised that the evidence for mediation would be greater. From the simulation study, we learned that indirect effect estimates may be attenuated if the mediator is measured infrequently. It is possible that annual measurements of lung function were not frequent enough for this process and that the estimated indirect effect was biased. Another potential source of bias is uncertainty in the mediator measurements. We do not believe this to be a substantial problem with the lung function measurements because of the standard laboratory procedures used and pre-planned measurement times at annual well visits. A further limitation is that some unexposed people later became exposed but their change in exposure status was not incorporated into the analysis. We chose not to censor them at the time they were diagnosed with CFRD because this would violate the assumption of non-informative censoring. The changing exposure status of some individuals may have biased the effect estimates and future work could include the specification of estimands for these two methods when the exposure is time-varying.

For the method of Aalen, we must posit an exposure separation. One possible mechanism for lung disease associated with diabetes is via a build-up of collagen in lung tissue leading to reduced elasticity.^37^ As this aspect is unlikely to affect survival other than via lung function, we may envision a split of this aspect away from the other effects of glucose intolerance to obtain a theoretical exposure separation. The further assumption of the method of Aalen that only the most recent measurement of lung function affects the hazard seems unlikely to hold as there is evidence that previous values of FEV1% are significant predictors of the hazard at a given time in addition to the most recent value.^38^ A causal interpretation for these analyses is also reliant upon the assumption of no unmeasured confounding. We attempted to control for confounding of the exposure-outcome and exposure-mediator relationships by adjusting for five baseline covariates which are known predictors of survival in CF but, as with all causal analyses using observational data, it is impossible to verify that confounding has been completely addressed. Controlling for confounding of the mediator-outcome relationship was via measurements of the two time-varying confounders (BMI and IV days) in the method of Vansteelandt. In the method of Aalen however, it was necessary to assume that sufficient control for confounding could be achieved using the baseline confounders. Because respiratory infections can be a time-varying common cause of both lung function and survival, this assumption is likely not valid and the method of Aalen results may be biased. In the method of Vansteelandt, a specific causal ordering of covariates and mediators was assumed where IV days and BMI measured at visit k could affect the FEV1% measured at visit k but not the reverse. This seems plausible for both because IV days quantifies the prior year’s days in hospital and there is evidence BMI affects lung function via its effect on respiratory musculature.^37^ In the analyses presented here, we adjusted for the baseline measurement of lung function to control for exposure-outcome and exposure-mediator confounding. We wish to stress the importance of adjusting for the baseline mediator measurement as an unadjusted analysis can produce very different results. For example, in a repeat analysis that differed only by not adjusting for baseline FEV1%, the indirect effect was estimated to be 0.96 at time t= 5 as opposed to 0.99 with adjustment. This equates to a tripling of the estimated proportion mediated.

Both of the methods studied here are valuable tools for mediation analysis in the setting of a survival outcome with a time-updated mediator. The method of Vansteelandt allows time-varying mediator-outcome confounding and can also be extended to multiple longitudinal mediators, a situation that will be common in clinical studies. The sample size required by the method of Vansteelandt to achieve the desired precision may be greater, particularly as the number of visit times increases because each regression is performed on the history of all of the covariates. At later visit times when there are more covariates in the model, there may be fewer observations due to fewer people having survived to that visit. An alternative evaluation technique for the method of Vansteelandt equation (5) is Monte Carlo integration but it requires jointly modelling the distribution of all variables. The method of Aalen offers fast computation times but is limited to the setting where time-varying confounding is believed not to exist. For some settings, it may be preferable to assume that the treatment or exposure can be split into separate biological components, as in the method of Aalen, than to contemplate a hypothetical intervention on the mediator. Also, further research into methods for assessing fit of these models would be helpful.

We have shown that both the method of Vansteelandt and the method of Aalen produce approximately unbiased results in a reference scenario consistent with both of their assumptions. Further, the method of Vansteelandt returned approximately unbiased effect estimates in a variety of scenarios where time-varying confounding was introduced. Both techniques rely on a number of assumptions to make causal statements and care should be taken with the interpretation of any analysis. The importance of discussions with experts on the clinical aspects of the data cannot be overstated.

## Supplemental Material

sj-pdf-1-smm-10.1177_09622802221107104 - Supplemental material for Methods of analysis for survival outcomes with time-updated mediators, with application to longitudinal disease registry data Supplemental material, sj-pdf-1-smm-10.1177_09622802221107104 for Methods of analysis for survival outcomes with time-updated mediators, with application to longitudinal disease registry data by Kamaryn T Tanner, Linda D Sharples, Rhian M Daniel and Ruth H Keogh in Statistical Methods in Medical Research

## Data Availability

The code for generating data used in the simulation study (section 4) is available from https://github.com/KamTan/MediationSimulation. SAS code for the method of Vansteelandt analysis is available in the online supplementary materials of Vansteelandt et al.^2^ and R code for the method of Aalen analysis is available in the online supporting material of Aalen et al.^3^ Although we are not permitted to make the UK CF Registry data public, researchers may apply for access to this dataset by completing a data request form found here: https://www.cysticfibrosis.org.uk/the-work-we-do/uk-cf-registry/apply-for-data-from-the-uk-cf-registry.
