# Supplementary material for: Methods of analysis for survival outcomes with time-updated mediators, with application to longitudinal disease registry data
Source: Stat Methods Med Res. 2022 Jun 16;31(10):1959–75. doi: 10.1177/09622802221107104 (PMC9523823; doi:10.1177/09622802221107104)
Supplement: sj-pdf-1-smm-10.1177_09622802221107104 - Supplemental material for Methods of analysis for survival outcomes with time-updated mediators, with application to longitudinal disease registry data [file sj-pdf-1-smm-10.1177_09622802221107104.pdf]

Supplemental Information for "Methods of analysis for survival outcomes with time-updated mediators, with application to longitudinal disease registry data"

Kamaryn T Tanner[1], Linda D Sharples[1], Rhian M Daniel[2], and Ruth H Keogh[1]

[1] *Dept of Medical Statistics, London School of Hygiene and Tropical Medicine, UK*
[2] *Div of Population Medicine, Cardiff University, UK*

May 2022

1 Simulation study: parameter values used

Table 1: Parameter values used to generate simulated data for the reference scenario. To scale the hazards so that the majority of survival times were < 4, the generated hazard was multiplied by a scaling factor, ξ.

Mediator Model		Hazard Model	
Param	Value	Param	Value
$\mu_M =$	$\begin{bmatrix} 2.9 \\ 0.0 \end{bmatrix}$	$\alpha_0 =$	0.75
$\Sigma_M =$	$\begin{bmatrix} 0.250 & -0.015 \\ -0.015 & 0.010 \end{bmatrix}$	$\alpha_{M_{\lfloor t \rfloor}} =$	$(0.3, 0.3, 0.3, 0.3)$
$\beta_{Z_0} =$	0.5	$\alpha_{Z_0} =$	0.35
$\beta_{A_t} =$	$(0, 2, 2, 2)$*	$\alpha_A =$	0.35**
		$\xi =$	0.35
* or $(0, 0, 0, 0)$ for NoIE scenario		** or 0 for NoDE scenario	

Table 2: Parameter values used to generate simulated data. For each scenario, only values that are different from the reference scenario reported in Table 1 are shown.

Type	Name	Values different from reference scenario
Reference		
	R1	$\alpha_{M_{\lfloor t \rfloor}} = (0.1, 0.2, 0.3, 0.4)$
	R2	$\alpha_{M_{\lfloor t \rfloor}} = (0.0, 0.75, 0.0, 0.0)$
	R3	$\alpha_{M_{\lfloor t \rfloor}} = (0.0, 0.0, 0.6, 0.6)$
Infrequent mediator measurements		
	F1	$\alpha_{M_{\lfloor t \rfloor}} = 0.3$ for $t = 0, 0.25, \ldots, 3.75$<br>$\beta_{A_t} = 2.0$ for $t = 0.25, \ldots, 3.75$
	F2	$\alpha_{M_{\lfloor t \rfloor}} = 0.3$ for $t = 0, 0.25, \ldots, 3.75$<br>$\beta_{A_t} = -2.0$ for $t = 0.25, \ldots, 3.75$
Time-varying covariates		
	All	$p_{Z_0 \mid A=1} = p_{Z_0 \mid A=0} = 0.5$<br>$\beta_{Z_0} = 0.25$<br>$\beta_{L_k} = (0.05,0.05,0.05,0.05)$<br>$\psi_{Z_0}=0$, $\psi_{M_{\lfloor t \rfloor}} = (0,0,0)$<br>$\psi_{A_t} = (0,0,0,0)$<br>$\alpha_0 = 0.5$, $\xi = 0.45$<br>$\alpha_A = 0.2$, $\alpha_{Z_0} = 0.1$<br>$\alpha_{M_{\lfloor t \rfloor}} = (0.1, 0.1, 0.1, 0.1)$<br>$\alpha_{L_{\lfloor t \rfloor}} = (0.01, 0.01, 0.01, 0.01)$
	L1	$\mu_L = \begin{bmatrix} 50 \\ 0.5 \end{bmatrix}$, $\Sigma_L = \begin{bmatrix} 100 & -4 \\ -4 & 4 \end{bmatrix}$
	L2	$\mu_L = \begin{bmatrix} 50 \\ 5 \end{bmatrix}$, $\Sigma_L = \begin{bmatrix} 100 & -4 \\ -4 & 4 \end{bmatrix}$
	L3	$\mu_L = \begin{bmatrix} 50 \\ 15 \end{bmatrix}$, $\Sigma_L = \begin{bmatrix} 100 & -4 \\ -4 & 4 \end{bmatrix}$
	L4	$\mu_L = \begin{bmatrix} 50 \\ 2.5 \end{bmatrix}$, $\Sigma_L = \begin{bmatrix} 100 & -10 \\ -10 & 25 \end{bmatrix}$
	L5	$\mu_L = \begin{bmatrix} 50 \\ 2.5 \end{bmatrix}$, $\Sigma_L = \begin{bmatrix} 100 & -10 \\ -10 & 100 \end{bmatrix}$
	L6	$\mu_L = \begin{bmatrix} 50 \\ 0.5 \end{bmatrix}$, $\Sigma_L = \begin{bmatrix} 100 & -4 \\ -4 & 4 \end{bmatrix}$<br>$\psi_{A_k} = (0,15,15,15)$

2 Simulation study: reference scenario

Table 3: Absolute bias of effect estimates for the reference scenario. Percent bias was $< 2\%$ in magnitude for all estimates. Results are shown at times corresponding to the 20th, 50th (median) and 80th percentile of event occurrence in each sub-scenario. The Monte Carlo Standard Error was < 0.005 for all estimates of absolute bias.

					Absolute bias					
			Truth			Aalen			Vansteelandt^{add}	
Events	Time	TE	DE	IE	TE	DE	IE	TE	DE	IE
Both Direct and Indirect Effects										
20%	1.21	0.93	0.97	0.96	0.00	0.00	0.00	0.00	0.00	0.00
50%	1.66	0.80	0.92	0.87	0.00	0.00	0.00	0.00	0.00	0.00
80%	2.45	0.62	0.84	0.74	0.00	0.00	0.00	0.01	0.01	0.01
No Direct Effect										
20%	1.24	0.95	1.00	0.95	0.00	0.00	0.00	0.00	0.00	0.00
50%	1.73	0.86	1.00	0.86	-0.01	0.00	0.00	0.00	0.00	0.00
80%	2.58	0.72	1.00	0.72	-0.01	0.00	-0.01	0.00	0.00	0.01
No Indirect Effect										
20%	1.26	0.97	0.97	1.00	0.00	0.00	0.00	0.00	0.00	0.00
50%	1.78	0.91	0.91	1.00	-0.01	0.00	-0.01	0.00	0.00	0.00
80%	2.66	0.81	0.82	1.00	-0.01	0.00	-0.02	0.00	0.00	0.00

Table 4: The empirical standard error and relative efficiency measured for the reference scenario. Efficiency is reported for the method of Aalen relative to the method of Vansteelandt, therefore, values greater than 1 indicate greater efficiency of the method of Aalen. Results are given for the time at which 50% of individuals had had an event.

	Empirical standard error								
		Aalen			Vansteelandt^{add}			Rel. Efficiency	
	TE	DE	IE	TE	DE	IE	TE	DE	IE
DE+IE	0.03	0.05	0.03	0.03	0.06	0.05	0.98	1.75	2.94
NoDE	0.03	0.05	0.03	0.03	0.06	0.05	0.99	1.55	2.43
NoIE	0.03	0.03	0.00	0.03	0.03	0.00	1.00	0.98	0.96

3 Simulation study: generation of truth

True values of the estimands were generated using a large simulated dataset as described in the main text. Figure 1 shows plots of the true values for the three survival curves for the reference scenario: $S_{A(1),M(1)}(t)$, $S_{A(0),M(0)}(t)$ and $S_{A(1),M(0)}(t)$ In the NoDE sub-scenario (upper-left), the total effect equals the indirect effect and, therefore, $S_{A(1),M(0)}(t) = S_{A(0),M(0)}(t)$. Similarly, in the NoIE sub-scenario (upper-right), because none of the total effect goes through the mediator, the total effect equals the direct effect and $S_{A(1),M(1)}(t) = S_{A(1),M(0)}(t)$. In the DE+IE sub-scenario (bottom-left), the dotted black line representing the survival curve $S_{A(1),M(0)}(t)$ is distinct from the other two curves indicating that both a direct and an indirect effect exist.

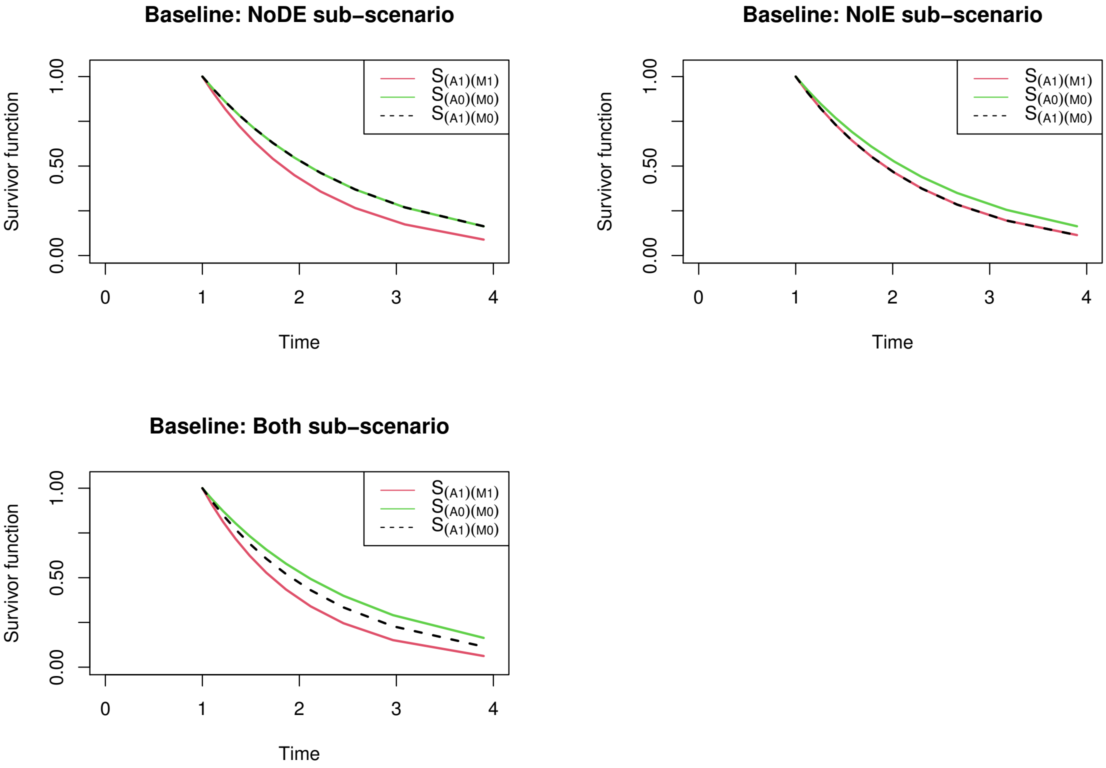

Figure 1: Survival curves based on a large simulated data set used to generate true values of the estimands. In the NoDE sub-scenario (upper-left), the total effect equals the indirect effect. In the NoIE sub-scenario (upper-right), there is no mediation present and DE=TE. In the DE+IE sub-scenario (bottom-left), both a direct and an indirect effect are present.

4 Simulation study: results from infrequent mediator measurement scenarios

In the scenario where A positively affected M, (F1), both methods produced biased results for both the direct and indirect effect estimates in the DE+IE and NoDE scenarios (Table 5). This led to a corresponding under-estimation of the DE. A similar pattern was seen in the NoDE sub-scenario. When the direction of the effect of A on M was reversed (F2), the bias in the estimates of DE and IE was even larger for both methods (Table 6). No bias was seen in the estimation of total effect for either method in either scenario. Because the frequency of the mediator measurements is inconsequential when there is no indirect effect, results from the NoIE sub-scenarios are not shown.

Table 5: Absolute bias (percent bias) of effect estimates for scenario F1 (A positively affects M). Bold indicates bias $\geq 5\%$. Event times were generated based on 4 mediator measurements per year but only annual mediator measurements were used in the analysis. Results are shown at times corresponding to the 20th, 50th and 80th percentile of event occurrence. The Monte Carlo Standard Error was < 0.006 for all estimates, for both methods.

					Absolute bias (percent bias)					
			Truth			Aalen			Vansteelandt^{add}	
Events	Time	TE	DE	IE	TE	DE	IE	TE	DE	IE
Both Direct and Indirect Effects										
20%	1.21	0.93	0.97	0.96	0.00 (0%)	0.00 (0%)	0.00 (0%)	0.00 (0%)	0.00 (0%)	0.00 (0%)
50%	1.66	0.80	0.92	0.87	0.00 (0%)	**-0.06 (-7%)**	**0.06 (7%)**	0.00 (0%)	**-0.06 (-6%)**	**0.06 (7%)**
80%	2.45	0.62	0.84	0.74	0.00 (0%)	**-0.12 (-15%)**	**0.13 (18%)**	0.00 (0%)	**-0.11 (-14%)**	**0.13 (18%)**
No Direct Effect										
20%	1.24	0.95	1.00	0.95	0.00 (0%)	0.00 (0%)	0.00 (0%)	0.00 (0%)	0.00 (0%)	0.00 (0%)
50%	1.72	0.86	1.00	0.86	0.00 (0%)	**-0.07 (-7%)**	**0.07 (8%)**	0.00 (0%)	**-0.07 (-7%)**	**0.07 (8%)**
80%	2.57	0.72	1.00	0.72	0.00 (0%)	**-0.16 (-16%)**	**0.15 (20%)**	0.00 (1%)	**-0.15 (-15%)**	**0.14 (20%)**

Table 6: Absolute bias (percent bias) of effect estimates for scenario F2 (A negatively affects M). Bold indicates bias $\geq 5\%$. Event times were generated based on 4 mediator measurements per year but but only annual mediator measurements were used in the analysis. Results are shown at times corresponding to the 20th, 50th and 80th percentile of event occurrence. The Monte Carlo Standard Error was < 0.010 for all estimates, for both methods.

					Absolute bias (percent bias)					
			Truth			Aalen			Vansteelandt^{add}	
Events	Time	TE	DE	IE	TE	DE	IE	TE	DE	IE
Both Direct and Indirect Effects										
20%	1.32	1.03	0.96	1.07	0.00 (0%)	0.01 (1%)	-0.01 (-1%)	0.00 (0%)	0.01 (1%)	-0.01 (-1%)
50%	1.93	1.08	0.89	1.21	0.00 (0%)	**0.11 (13%)**	**-0.13 (-11%)**	0.00 (0%)	**0.11 (13%)**	**-0.13 (-11%)**
80%	2.88	1.18	0.79	1.48	0.01 (0%)	**0.22 (27%)**	**-0.31 (-21%)**	0.00 (0%)	**0.20 (25%)**	**-0.29 (-19%)**
No Direct Effect										
20%	1.36	1.08	1.00	1.08	0.00 (0%)	0.02 (2%)	-0.02 (-2%)	0.00 (0%)	0.02 (2%)	-0.02 (-2%)
50%	2.04	1.24	1.00	1.24	0.00 (0%)	**0.14 (14%)**	**-0.15 (-12%)**	0.00 (0%)	**0.14 (14%)**	**-0.15 (-12%)**
80%	3.01	1.52	1.00	1.53	0.00 (0%)	**0.31 (31%)**	**-0.35 (-23%)**	0.00 (0%)	**0.28 (28%)**	**-0.33 (-21%)**

5 Simulation study: results from time-varying confounder scenarios

Figure 2 shows the values of L for 60 simulated individuals for each scenario with time-varying confounding, L1-L6. The top row represents scenarios with differing mean random slope, μ_{l0}. The bottom row (left and centre) plots L when the standard deviation of the random slope is increased and the bottom-right figure corresponds to the scenario where there is a strong effect of A on L.

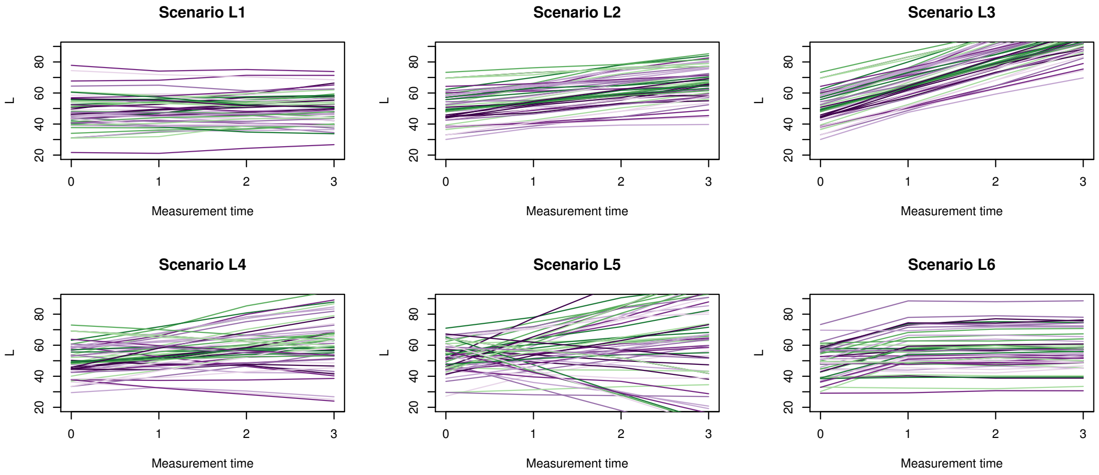

Figure 2: Values of the time-varying confounder L for 60 simulated individuals in the time-varying confounding scenarios L1-L6. Top row: mean random slope used to generate L is 0.5 (left), 5.0 (centre), 15.0 (right). Bottom row: standard deviation of the random slope used to generate L is 5.0 (left) or 10.0 (centre) and the scenario when the exposure affects L (right).

Table 7: Absolute bias of effect estimates for scenario L1 with a time-varying covariate with mean random slope = 0.5 that is not affected by the exposure. Percent bias was $\leq 1\%$ for all estimates. Results are shown at times corresponding to the 20th, 50th (median) and 80th percentile of event occurrence in each sub-scenario. The Monte Carlo Standard Error was < 0.005 for all estimates of absolute bias.

					Absolute bias					
			Truth			Aalen			Vansteelandt^{add}	
Events	Time	TE	DE	IE	TE	DE	IE	TE	DE	IE
Both Direct and Indirect Effects										
20%	1.26	0.96	0.98	0.98	0.00	0.00	0.00	0.00	0.00	0.00
50%	1.78	0.87	0.93	0.93	0.00	0.00	0.00	0.00	0.00	0.00
80%	2.67	0.74	0.86	0.86	0.00	0.01	0.00	0.00	0.00	0.01
No Direct Effect										
20%	1.28	0.98	1.00	0.98	0.00	0.00	0.00	0.00	0.00	0.00
50%	1.85	0.93	1.00	0.93	0.00	0.00	0.00	0.00	0.00	0.00
80%	2.76	0.85	1.00	0.85	0.00	0.01	0.00	0.00	0.01	0.01
No Indirect Effect										
20%	1.28	0.97	0.97	1.00	0.00	0.00	0.00	0.00	0.00	0.00
50%	1.84	0.93	0.93	1.00	0.00	0.00	0.00	0.00	0.00	0.00
80%	2.76	0.85	0.85	1.00	0.00	0.00	0.00	0.00	0.00	0.00

Table 8: Absolute bias of effect estimates for scenario L2 with a time-varying covariate with mean random slope = 5.0 that is not affected by the exposure. Percent bias was $\leq 1\%$ for all estimates. Results are shown at times corresponding to the 20th, 50th (median) and 80th percentile of event occurrence in each sub-scenario. The Monte Carlo Standard Error was < 0.005 for all estimates of absolute bias.

					Absolute bias					
			Truth			Aalen			Vansteelandt^{add}	
Events	Time	TE	DE	IE	TE	DE	IE	TE	DE	IE
Both Direct and Indirect Effects										
20%	1.25	0.96	0.98	0.98	0.00	0.00	0.00	0.00	0.00	0.00
50%	1.77	0.87	0.93	0.93	0.00	0.00	0.00	0.00	0.00	0.00
80%	2.64	0.75	0.86	0.87	0.00	0.01	-0.01	0.00	0.01	0.01
No Direct Effect										
20%	1.28	0.98	1.00	0.98	0.00	0.00	0.00	0.00	0.00	0.00
50%	1.84	0.93	1.00	0.93	0.00	0.00	0.00	0.00	0.00	0.00
80%	2.74	0.86	1.00	0.85	0.00	0.01	0.00	0.00	0.00	0.01
No Indirect Effect										
20%	1.28	0.97	0.98	1.00	0.00	0.00	0.00	0.00	0.00	0.00
50%	1.84	0.93	0.93	1.00	0.00	0.00	0.00	0.00	0.00	0.00
80%	2.74	0.86	0.85	1.00	0.00	0.01	0.00	0.01	0.01	0.00

Table 9: Absolute bias of effect estimates for scenario L3 with a time-varying covariate with mean random slope = 15.0 that is not affected by the exposure. Percent bias was $\leq 1\%$ for all estimates. Results are shown at times corresponding to the 20th, 50th (median) and 80th percentile of event occurrence in each sub-scenario. The Monte Carlo Standard Error was < 0.005 for all estimates of absolute bias.

					Absolute bias					
		Truth			Aalen			Vansteelandt^{add}		
Events	Time	TE	DE	IE	TE	DE	IE	TE	DE	IE
Both Direct and Indirect Effects										
20%	1.24	0.96	0.98	0.98	0.00	0.00	0.00	0.00	0.00	0.00
50%	1.74	0.87	0.93	0.94	0.00	0.00	0.00	0.00	0.00	0.00
80%	2.57	0.76	0.87	0.87	0.00	0.01	0.00	0.00	0.01	0.00
No Direct Effect										
20%	1.27	0.98	1.00	0.98	0.00	0.00	0.00	0.00	0.00	0.00
50%	1.81	0.93	1.00	0.93	0.00	0.00	0.00	0.00	0.00	0.00
80%	2.67	0.86	1.00	0.86	0.00	0.01	0.00	0.00	0.01	0.01
No Indirect Effect										
20%	1.27	0.98	0.98	1.00	0.00	0.00	0.00	0.00	0.00	0.00
50%	1.81	0.93	0.93	1.00	0.00	0.00	0.00	0.00	0.00	0.00
80%	2.67	0.86	0.86	1.00	0.00	0.01	0.00	0.01	0.01	0.00

Table 10: Absolute bias of effect estimates for scenario L4 with a time-varying covariate that is not affected by the exposure and has a moderate random slope variance. Percent bias is shown beneath the absolute bias in parentheses. Results are shown at times corresponding to the 20th, 50th (median) and 80th percentile of event occurrence in each sub-scenario. The Monte Carlo Standard Error was < 0.005 for all estimates of absolute bias

					Absolute bias (percent bias)					
			Truth			Aalen			Vansteelandt^{add}	
Events	Time	TE	DE	IE	TE	DE	IE	TE	DE	IE
Both Direct and Indirect Effects										
20%	1.26	0.96	0.98	0.98	0.00	0.00	0.00	0.00	0.00	0.00
					(0%)	(0%)	(0%)	(0%)	(0%)	(0%)
50%	1.78	0.87	0.93	0.93	0.00	0.01	-0.01	0.00	0.00	0.00
					(0%)	(1%)	(-1%)	(0%)	(0%)	(0%)
80%	2.65	0.74	0.86	0.86	0.00	0.03	-0.02	0.00	0.01	0.00
					(0%)	(3%)	(-3%)	(0%)	(1%)	(0%)
No Direct Effect										
20%	1.28	0.98	1.00	0.98	0.00	0.00	0.00	0.00	0.00	0.00
					(0%)	(0%)	(0%)	(0%)	(0%)	(0%)
50%	1.84	0.93	1.00	0.93	0.00	0.01	-0.01	0.00	0.00	0.00
					(0%)	(1%)	(-1%)	(0%)	(0%)	(0%)
80%	2.75	0.85	1.00	0.85	0.00	0.03	-0.02	0.00	0.00	0.01
					(0%)	(3%)	(-3%)	(0%)	(0%)	(1%)
No Indirect Effect										
20%	1.28	0.97	0.98	1.00	0.00	0.00	0.00	0.00	0.00	0.00
					(0%)	(0%)	(0%)	(0%)	(0%)	(0%)
50%	1.84	0.93	0.93	1.00	0.00	0.00	0.00	0.00	0.00	0.00
					(0%)	(0%)	(0%)	(0%)	(0%)	(0%)
80%	2.75	0.85	0.85	1.00	0.00	0.01	0.00	0.00	0.01	0.00
					(0%)	(1%)	(0%)	(1%)	(1%)	(0%)

Table 11: Absolute bias of effect estimates for scenario L5 with a time-varying covariate that is not affected by the exposure and has a large random slope variance. Percent bias is shown beneath the absolute bias in parentheses. Bold indicates bias $\geq 5\%$. Results are shown at times corresponding to the 20th, 50th and 80th percentile of event occurrence in each sub-scenario. The Monte Carlo Standard Error was < 0.005 for all estimates of absolute bias.

					Absolute bias (percent bias)					
			Truth			Aalen			Vansteelandt^{add}	
Events	Time	TE	DE	IE	TE	DE	IE	TE	DE	IE
Both Direct and Indirect Effects										
20%	1.26	0.96	0.98	0.98	0.00	0.01	-0.01	0.00	0.00	0.00
					(0%)	(1%)	(-1%)	(0%)	(0%)	(0%)
50%	1.77	0.87	0.93	0.93	0.00	0.02	-0.02	0.00	0.00	0.00
					(0%)	(2%)	(-2%)	(0%)	(0%)	(0%)
80%	2.64	0.75	0.86	0.86	-0.00	**0.07**	**-0.07**	0.00	0.01	0.00
					(0%)	**(9%)**	**(-8%)**	(0%)	(1%)	(0%)
No Direct Effect										
20%	1.28	0.98	1.00	0.98	0.00	0.01	-0.01	0.00	0.00	0.00
					(0%)	(1%)	(-1%)	(0%)	(0%)	(0%)
50%	1.83	0.93	1.00	0.93	0.00	0.02	-0.02	0.00	0.00	0.00
					(0%)	(2%)	(-2%)	(0%)	(0%)	(0%)
80%	2.73	0.86	1.00	0.85	0.00	**0.09**	**-0.07**	0.00	0.00	0.01
					(0%)	**(9%)**	**(-8%)**	(0%)	(0%)	(1%)
No Indirect Effect										
20%	1.28	0.98	0.98	1.00	0.00	0.00	0.00	0.00	0.00	0.00
					(0%)	(0%)	(0%)	(0%)	(0%)	(0%)
50%	1.83	0.93	0.93	1.00	0.00	0.00	0.00	0.00	0.00	0.00
					(0%)	(0%)	(0%)	(0%)	(0%)	(0%)
80%	2.73	0.86	0.85	1.00	0.00	0.00	0.00	0.00	0.01	0.00
					(0%)	(1%)	(0%)	(0%)	(1%)	(0%)

Table 12: Absolute bias of effect estimates for scenario L6 with a time-varying covariate that is affected by the exposure. Percent bias is shown beneath the absolute bias in parentheses. Bold indicates bias $\geq$ 5%. Results are shown at times corresponding to the 20th, 50th (median) and 80th percentile of event occurrence in each sub-scenario. The Monte Carlo Standard Error was < 0.005 for all estimates of absolute bias.

					Absolute bias (percent bias)					
			Truth			Aalen			Vansteelandt^{add}	
Events	Time	TE	DE	IE	TE	DE	IE	TE	DE	IE
Both Direct and Indirect Effects										
20%	1.27	0.94	0.98	0.96	0.00	-0.02	0.02	0.00	0.00	0.00
					(0%)	(-2%)	(2%)	(0%)	(0%)	(0%)
50%	1.81	0.82	0.93	0.88	0.00	**-0.05**	**0.05**	0.00	0.00	0.00
					(0%)	**(-5%)**	**(6%)**	(0%)	(0%)	(0%)
80%	2.71	0.66	0.86	0.76	0.00	**-0.09**	**0.09**	0.00	0.01	0.01
					(0%)	**(-11%)**	**(12%)**	(0%)	(1%)	(1%)
No Direct Effect										
20%	1.32	0.97	1.00	0.97	0.00	0.00	0.00	0.00	0.00	0.00
					(0%)	(0%)	(0%)	(0%)	(0%)	(0%)
50%	1.93	0.92	1.00	0.92	0.00	0.00	0.00	0.00	0.00	0.00
					(0%)	(0%)	(0%)	(0%)	(0%)	(0%)
80%	2.88	0.84	1.00	0.84	0.00	0.00	0.00	0.00	0.00	0.01
					(0%)	(0%)	(0%)	(0%)	(0%)	(1%)
No Indirect Effect										
20%	1.29	0.95	0.97	0.98	0.00	-0.02	0.02	0.00	0.00	0.00
					(0%)	(-2%)	(2%)	(0%)	(0%)	(0%)
50%	1.88	0.87	0.92	0.94	0.00	**-0.05**	**0.06**	0.00	0.00	0.00
					(0%)	**(-6%)**	**(6%)**	(0%)	(0%)	(0%)
80%	2.80	0.75	0.85	0.89	0.00	**-0.10**	**0.11**	0.00	0.00	0.00
					(0%)	**(-11%)**	**(13%)**	(0%)	(1%)	(0%)

6 Simulation study: impact of conditioning on survival to first mediator measurement

Because we chose to use a population restricted to those who had survived to the first mediator measurement in our main analysis, we also assessed the impact of not estimating effects conditional on survival to the first mediator measurement using the method of Vansteelandt to analyse the reference scenario data with and without individuals having events prior to the first mediator measurement.

We compared estimates from an analysis of the reference scenario conditional on survival to t =1 with an unconditional analysis using the method of Vansteelandt. Figure 3 shows the effect estimates for the conditional analysis (orange) and the unconditional analysis (green). Because there were no events prior to t =1 in the conditional analysis, the TE, DE and IE were all equal to 1.0 at t =1. In contrast, in the unconditional analysis, some events have occurred prior to the first visit time and, because the exposure negatively affects survival in our simulation, the TE and DE estimates were less than one at t =1. Over time, the estimated DE and TE from the unconditional analysis continues to be lower than the corresponding estimates in the conditional analysis. However, as shown in Figure 3-top left, the indirect effect estimates coincide between the two analyses. Although proportion mediated was not studied in detail here, it is a commonly reported measure and Figure 3 (bottom right) shows that the difference in estimated proportion mediated is large between the conditional and unconditional analyses. At t =1.5, for example, the conditional analysis suggests that approximately 60% of the total effect is mediated but the unconditional analysis suggests that less than 40% is mediated.

We found that the estimates of TE and DE will differ between an analysis conditional on survival to the first mediator measurement and an unconditional analysis but the estimates of IE will be the same. As long as there is a non-zero DE and TE and there are events prior to t =1, their estimates will necessarily be different between the conditional and unconditional analyses. Because the IE cannot be estimated prior to measuring the mediator, these estimates coincide. Therefore, if the goal is to quantify the IE only, performing an analysis conditional on survival to the first mediator measurement should not change the results. If the goal is to estimate the proportion mediated, these two analyses will yield different results because the denominator of the proportion mediated is the total effect.

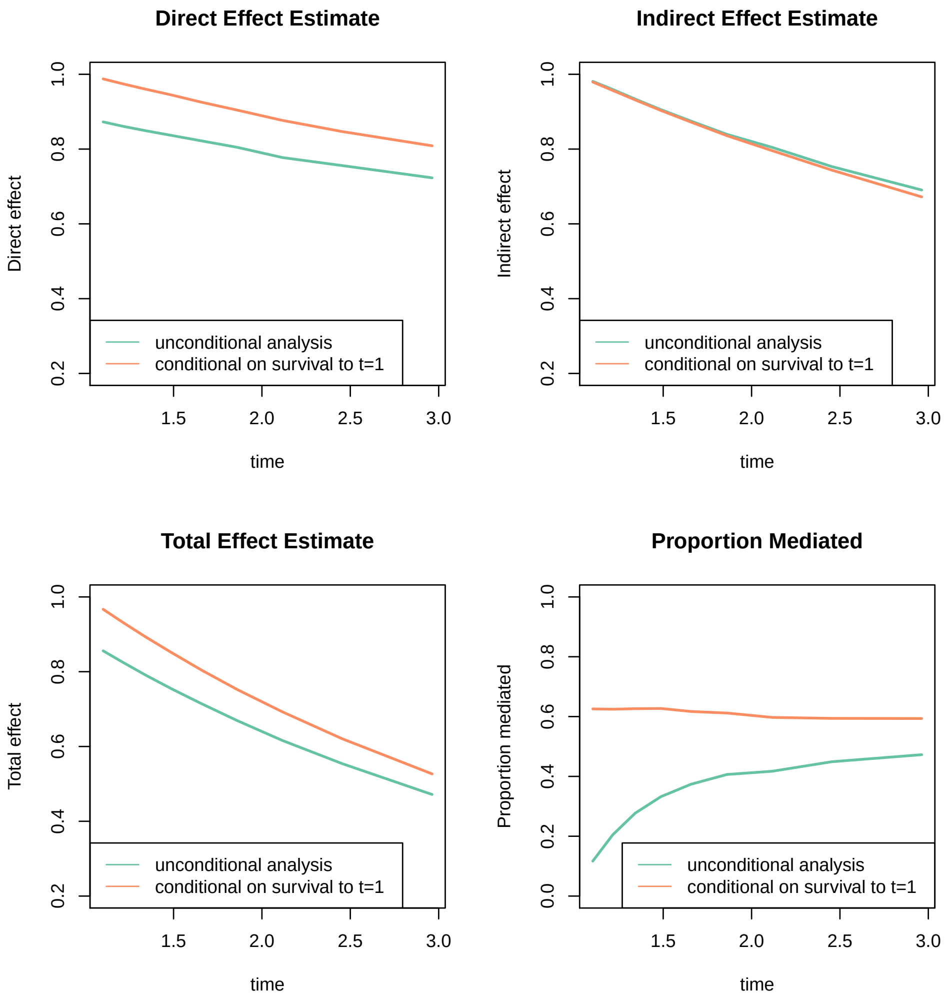

Figure 3: Comparison of effect estimates from the method of Vansteelandt for an analysis restricted to individuals who survive to the first mediator measurement (orange) versus an analysis including all individuals (green). Estimates are plotted from the time at which 10% of events had occurred in the conditional analysis ($t = 1.1$) to the time at which 90% of events had occurred ($t = 2.96$).

7 Application to CFRD: dataset creation

To construct the mediation analysis dataset, we vertically stacked age-specific datasets for ages 18-50 years. We first assumed that all data measurements were taken at integer-valued ages, a, using data from the annual review that most closely preceded each individual's a^{th} birthday. The age-specific dataset for age a comprises individuals who are at risk at age a and have either been diagnosed with CFRD within the past year (the exposed) or have not been diagnosed with CFRD (the unexposed). Individuals contribute data when unexposed to multiple age-specific datasets but will only contribute once as an exposed person. Figure 4 illustrates the creation of age-specific sets of data for two hypothetical individuals A and B from ages 23 to 26 where FEV1 is the repeatedly-measured mediator. In this example, the earliest data available for A and B is at age 23 so we use this data for baseline measurements in the age a =24 dataset to ensure proper causal ordering from $Z_0 \rightarrow A \rightarrow M_1$. For example, the age a =24 dataset for person A is created by setting the start time to 0 at age 24, and then adding start and stop times to indicate the range of times (where age 24 equals time 0) over which the mediator measurement is valid. The age a is added to each row of the dataset for adjustment. An event indicator is set to 1 to indicate an event occurred at the associated stop time and the CFRD indicator will be 1 in the age-specific dataset in which the individual was diagnosed. A contributes data to the ages a =24, 25, 26 age-specific datasets. Individual B was diagnosed with CFRD at age 25, therefore, B does not contribute to the a =26 dataset because we only use data from the first age at which a diagnosis of CFRD occurs. The age-specific data for all individuals is then vertically stacked to form one analysis dataset.

Original Data:

id	age	CFRD	FEV1	sex	age at event	event
A	23	0	43.5	M	27.0	0
A	24	0	56.6	M	27.0	0
A	25	0	48.3	M	27.0	0
A	26	0	38.9	M	27.0	0
B	23	0	43.3	F	26.2	1
B	24	0	36.9	F	26.2	1
B	25	1	29.5	F	26.2	1
B	26	1	35.0	F	26.2	1

Analysis Data:

					Exposure	Mediator	Baseline Confounders	
id	age	start	stop	event	CFRD	FEV1	sex	FEV1 at baseline
A	24	0.0	1.0	0	0	56.56	M	43.5
A	24	1.0	2.0	0	0	48.33	M	43.5
A	24	2.0	3.0	0	0	38.94	M	43.5
A	25	0.0	1.0	0	0	48.33	M	56.6
A	25	1.0	2.0	0	0	38.94	M	56.6
A	26	0.0	1.0	0	0	38.94	M	48.3
B	24	0.0	1.0	0	0	36.90	F	43.3
B	24	1.0	2.0	0	0	29.51	F	43.3
B	24	2.0	2.2	1	0	34.99	F	43.3
B	25	0.0	1.0	0	1	29.51	F	36.9
B	25	1.0	1.2	1	1	34.99	F	36.9

Not used, only incident cases of CFRD are considered

Figure 4: Construction of the mediation analysis dataset. On the left, a table representing the raw data, formatted with one row per person per integer age. On the right, a table showing the analysis dataset with age-specific data for each individual. This data is formatted with start and stop times indicating the valid time for the mediator measurement. Individual A contributes 3 age-specific sets of data (ages= 24, 25, 26) and individual B contributes 2 age-specific sets of data (ages=24, 25). Only people newly diagnosed with CFRD and people without CFRD contribute data.
